# Reduced specificity and increased overgenerality of autobiographical memory persist as cognitive vulnerabilities in remitted major depression: A meta‐analysis

**DOI:** 10.1002/cpp.2786

**Published:** 2022-10-04

**Authors:** David John Hallford, Danielle Rusanov, Joseph J. E. Yeow, Tom Joseph Barry

**Affiliations:** ^1^ School of Psychology Deakin University Geelong Victoria Australia; ^2^ School of Psychology Deakin University Burwood Victoria Australia; ^3^ Faculty of Social Sciences University of Hong Kong Pok Fu Lam Hong Kong

**Keywords:** major depression, memory specificity, meta‐analysis, overgeneral memory, remission

## Abstract

Difficulty in accessing specific memories, referred to as reduced memory specificity or overgeneral memory (OGM), has been established as a marker of clinical depression. However, it is not clear if this deficit persists following the remission of depressive episodes. The current study involved a systematic review and meta‐analysis of empirical studies with the aim of establishing whether remitted depression was associated with retrieving fewer specific and more overgeneral autobiographical memories. Seventeen studies were identified as eligible. The results indicated that people with remitted depression recalled fewer specific memories (*k* = 15; *g* = −0.314, 95% CI [−0.543; −0.085], *z* = −2.69, *p* = .007) and more categoric memories (*k* = 9; *g* = 0.254, 95% CI [0.007; 0.501], *z* = 2.02, *p* = .043) compared to people who had never been depressed. Given these deficits have elsewhere been shown to be prognostic of future depressive symptoms, these findings suggest that reduced memory specificity/overgeneral memory persists following remission and may be a risk factor for future episodes of depression in those that are in remission. The findings are discussed in terms of how this knowledge might influence clinical understanding of relapse prevention and maintenance of remission in those with a history of depression.

**Key Practitioner Message**

- Difficulties recalling specific personal memories are a marker of depression.
- This deficit persists after remission relative to those with no history of depression.
- Poorer autobiographical memory may be a vulnerability for future episodes.
- Improving autobiographical memory specificity could protect against relapse.

## 1 INTRODUCTION

Autobiographical memories refer to information about one's personal past experiences, with specific memories referring to discrete event‐level experiences, no longer than a day in duration (e.g. *a long walk in a local park with two friends*). Difficulty in accessing specific memories, referred to as reduced memory specificity or overgeneral memory, has been established as a cognitive marker of depression (Liu et al., 2013; Williams, 1992) and other mental illnesses (Barry, Hallford, & Takano, 2021; van Vreeswijk & de Wilde, 2004). On failing to retrieve specific memories, people may instead recall non‐specific events, including recurrent events or *categories* of events (e.g. my daughters' birthdays or birthday parties), or events that lasted for *extended* periods of time (e.g. when I was a teenager I was very active). Difficulty in recalling specific memories is a predictor of future depressive symptoms, independent of depressive symptoms at baseline (Hallford, Rusanov, et al., 2021). This effect is significantly stronger in those that are already clinically depressed. This suggests that reduced memory specificity/overgeneral memory is both a risk factor for depressive symptoms in general and a relatively stronger maintaining factor when people do then experience clinical depression. This is perhaps unsurprising given that retrieving specific information about one's past experiences is implicated in adaptive processes such as problem‐solving (Hallford, Noory, & Mellor, 2018), planning and decision‐making (Dalgleish & Werner‐Seidler, 2014), up‐regulating anticipatory pleasure for future events (Hallford et al., 2020; Painter & Kring, 2016) and maintaining social support (Barry, Vinograd, et al., 2019; Chiu et al., 2019).

Although deficits in memory specificity have been well established in clinical depression, it is less clear whether they are *caused* by depression, or processes associated with depression, such rumination or issues with executive functioning (Williams et al., 2007). Further, it is unknown whether or not this deficit persists following the remission of clinical depression. That is, when people remit from episodes of major depression, are they still more likely to retrieve non‐specific memories relative to people who have not been depressed? If these difficulties do persist, then difficulties retrieving specific memories represent not only a symptom of depression but also a marker of vulnerability to future episodes. Studies to date provide conflicting answers to this question. For example, Kuyken and Dalgleish (2011) and Mackinger et al. (2000) found large differences in memory specificity between people in remission from depression and healthy control participants with no history of depression. In contrast, other studies, such as those by Crane et al. (2007) and Park et al. (2002) found small, non‐significant differences. There may be a number of reasons for this heterogeneity in findings. For instance, smaller samples less accurately estimate population differences given the greater variance in sampling distribution. They also have less statistical power to detect small effects, meaning researchers may draw false‐negative conclusions when the ‘true’ effects are only modest in size. It could also be that there are differences in studies on current/residual depressive symptoms between those in remission from depression and those with no history of depression. Potentially, group differences in memory specificity/overgenerality could be accounted for by these current/residual depressive symptoms, rather than attributed to an effect of historical depressive symptoms. Another consideration might be the number of previous episodes of depression that people have experienced, with a greater number of previous episodes potentially entrenching differences in memory specificity/overgenerality relative to those without a history of depression.

In addition to depression being a highly prevalent disorder (Lim et al., 2018), it is also a recurring illness, with estimates that 50% of people experience multiple episodes or a more chronic course of illness (Eaton et al., 2008). More clarity around risk factors for relapse, such as low memory specificity, will be invaluable in identifying who is more likely to become unwell and to inform interventions designed to maintain remission of depression. Although one previous review on cognitive abilities in remitted depression reported on a very small number of studies assessing specificity and overgenerality (Semkovska et al., 2019), the criteria for a healthy comparator group did not include ruling out a history of depression. Therefore, one cannot assume that the observed effects (which were around the moderate range) are a result of that independent variable.

### 1.1 Objectives

Against this background, the aim of this study was to examine whether people who are in remission from clinical depression show reduced specificity and increased overgeneral retrieval of personal memories relative to people that have never experienced clinical depression. To achieve this, a systematic review of the literature was conducted, and the studies from this review were meta‐analysed to estimate if whether, and to what extent, there were differences between these two populations across the breadth of available literature. Potential moderator variables (discussed below) were also analysed to help determine if there were specific factors that may predict any observed differences. Given previous findings, it was hypothesized that people with a history of depression would have significantly reduced memory specificity and significantly increased overgeneral memory relative to those who had never been depressed.

## 2 METHOD

The study procedure was pre‐registered within the PROSPERO database (ID: CRD42020203509). No deviation was made except for the addition of some moderator variables (see below) given the studies included in the review had these affordances. The data and scripts used for analysis in this study are open access and available at https://osf.io/bfcyj/.

### 2.1 Information sources and search strategy

**FIGURE 1:**
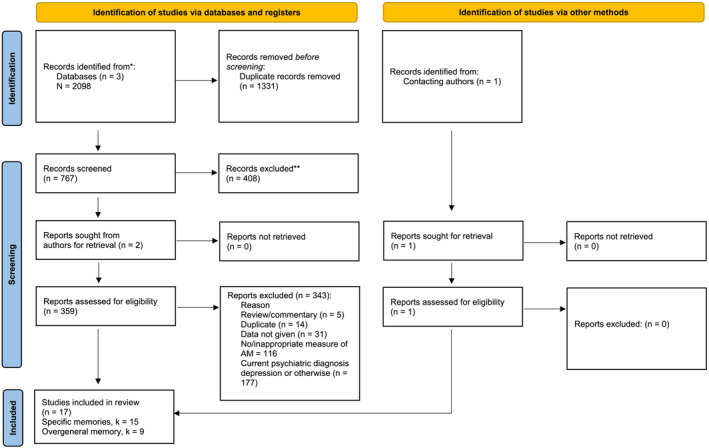
PRIMSA flowchart for the systematic search.

The search strategy involved using the search engines ProQuest and Ovid to search for keywords on the Embase, PsycARTICLES and PsycINFO databases. The current systematic search was nested within a larger systematic search, which aimed to collect all articles that examined memory specificity in clinical and non‐clinical samples (Barry, Hallford, & Takano, 2021). The included keywords related to psychiatric disorders, including depression, *autobiographical memory* and combinations of either *specificity* or *overgeneral*. A full list of search terms can be viewed in Data S2. From this broader search, only articles relating to depression and remitted depression were of interest. Search terms were included to try and examine processes that might explain any group differences (e.g., *rumination*, *brooding*, *executive function*, *verbal fluency*, *problem‐solving*, etc.; see Williams, 2006 for a review of these processes). However, within the present analysis, there were too few studies involving samples of people with remitted depression for a systematic review or meta‐analysis of these factors, and so this was not attempted. Following the database search, review articles including systematic reviews and meta‐analyses were examined for relevant citations that were missed in the first iteration. Relevant experts within the field were contacted and consulted for additional data, and the authors' own collection of unpublished data was considered where appropriate. The search was conducted on 18 April 2021 and request for studies shortly thereafter. See Figure 1 for a flow chart and Data S1 for a completed 2020 updated PRISMA checklist for reporting standards (Page et al., 2021).

### 2.2 Eligibility criteria

To be included, studies needed to be written in English, report original empirical data and contain two groups of participants: one defined by a history of clinical depression but currently in remission and another with no historical or current clinical depression. This, therefore, would not include studies that compared people with remitted and current depression (e.g. Brittlebank et al., 1993). Classification of a historical diagnosis of depression and the ruling out of current diagnosis of depression was to be performed using criteria from the Diagnostic and Statistical manuals (DSM) or International Classification of Diseases (ICD), either through clinical interview or a structured interview with a trained clinician. Although memory specificity can be assessed using a number of different measures, in order to reduce heterogeneity between the studies, an inclusion criterion was use of the autobiographical memory test (AMT) (Williams & Broadbent, 1986) to assess memory specificity/overgenerality. The AMT uses a protocol, whereby participants are provided with a list of cue words one by one and are asked to provide autobiographical memories, which are then coded in terms of how specific or non‐specific they are. It was expected that the majority of studies within this area of research would have used the AMT. It was further specified that studies could report AMT outcomes in terms of the number or proportion of specific memories or the number or proportion of general or non‐specific memories, and these would be meta‐analysed separately. A specific memory was to be defined as a discrete event lasting less than 24 h (*when I walked my dog last Friday*). A general memory could refer either to a categorical event (i.e. a memory that occurred on multiple occasions, *when I walk my dog*) or an extended event (i.e. a memory that occurred over a period of time longer than 24 h, *when I owned a dog*) or could be presented as a sum of both types of these events. Studies must have reported adequate data to be included in analyses (i.e. means and standard deviations of AMT scores, or other summary statistics from which mean and standard deviations can be derived, and study sample sizes).

### 2.3 Data extraction and handling

Two trained research assistants (co‐authors/and/) conducted the search separately. One research assistant then extracted all data initially, and the second research assistant then extracted all the data again checking for discrepancies. The first author (/) then checked the data, and any remaining disparities were resolved through discussion. We extracted the number of participants in each group within a given study, their mean age, the proportion of women and the diagnostic tool used to assess for major depression, measure of depressive symptom severity, number of previous episodes of depression, number of cues used, cue valence and duration for cue response. Where a study explicitly reported the education levels and ethnicity of participants, this was also extracted.

The mean and standard deviation scores for both the clinical and control group for the measure depression symptom severity used within each study were extracted. Although studies were selected on the basis that neither of the groups of participants being compared had clinical depression, nonetheless there may have been differences in depressive symptoms that might account for group differences in specific or general memories. Therefore, a standardized mean difference for severity scores between groups for each study was computed, and this variable was assessed as a potential moderator through meta‐regression. We extracted the mean and standard deviation for the number or proportion of specific and general memories retrieved across cues on the AMT. Where this information was available for different cue valences, this was also extracted so these could be analysed separately. We also extracted the duration of time participants were given to recall each memory following cue presentation, whether responses could be given verbally or otherwise and the number of cues given to participants.

### 2.4 Analytic strategy

Random‐effects meta‐analyses with maximum likelihood estimators were conducted using the meta package (Balduzzi et al., 2019) in R statistical software 4.0.3 (R Core Team (2020), 2020) for specific and overgeneral memories separately, using Hedges' *g* as the effect size. Forest plots were used to graphically depict the overall effect size and 95% confidence interval and prediction interval, as well as the individual study point estimates and 95% confidence intervals. Between‐study effect size heterogeneity was reported in terms of *Q*, *τ* ^2^ and *I* ^*2*^. The *Q* statistic provides an indication that factors outside of sampling error account for effect size estimate variation (Lipsey & Wilson, 2001). The *τ* ^2^ statistic indicates the absolute value of the true variance. The power of the *Q* statistic to detect statistically significant differences is based on the number of studies used in the meta‐analysis. In contrast, the *I* ^2^ index does not rely on statistical significance and is instead a percentage of total variation in a set of effect sizes that is due to heterogeneity between studies rather than chance (Higgins & Thompson, 2002). These analyses were conducted for overall effect sizes across cue types and for each cue valence separately.

Regarding potential moderators of any observed heterogeneity, we tested for differences in depressive symptom severity between the group by using the effect size estimated from means and SDs, age, the proportion of women (to assess for the influence of gender), number of cues used in the AMT, the duration of time given for retrieval on the AMT, the year of publication and sample size. Given that extreme scores might unduly influence an overall effect, we identified outliers as any study for which its confidence intervals did not overlap with the confidence interval of the pooled effect size. Sensitivity analyses were conducted by removing these studies and then conducting the analyses again to assess for changes in the overall effect.

### 2.5 Risk of bias

To assess for bias, we used several different methods. At the study level, sources of bias were assessed by auditing whether (1) the study involved randomisation between and within the study tasks (e.g. were cue words presented in a fixed or random order), (2) participants' group allocation was concealed from them, (3) participants and personnel were blind to the nature of the study, (4) the coders for the autobiographical memory task were blind to participants' group designation and the nature of the study during coding, (5) there was evidence for incomplete outcome reporting or for the contrary such as with pre‐registration, (5) particular participants were included in the study, but were omitted from analyses for unclear reasons, and (6) scores for particular measures were included in the study, but were selectively omitted from the final report. If these potential sources of bias were observed, then *a high risk of bias* was noted. If the study did not include enough information to assess clearly whether this was a potential source of bias, *some concern* was noted. If the study explicitly noted the steps taken to manage the bias mentioned, then a *low risk of bias* was noted. To assess publication bias in the sample of studies, we generated funnel plots, on which effect size estimates were plotted on the x‐axis, and the inverse of their standard error on the y‐axis. Plots resemble a funnel, with less precise estimates at the base of the funnel and estimates with the smallest standard errors at the top. If there is no publication bias, the funnel plot will, hypothetically, be symmetrical. However, missing studies suppressed by publication bias may cause noticeable asymmetry in a funnel plot. Egger's test (Egger et al., 1997) was used as a statistical test for funnel plot asymmetry, with a significant *p*‐value indicative of funnel plot asymmetry, and therefore publication bias. The trim‐and‐fill procedure was conducted, which estimates ‘missing studies’ until there is funnel plot symmetry and produces an adjusted effect size. Notably, this procedure may not produce reliable results in circumstances where there is notable between‐study heterogeneity. A *p‐*curve analysis was also used to assess the potential of *p‐*hacking, that is, selective presentation or analysis of data motivated to attain a *p*‐value below an alpha of .05 (Simonsohn et al., 2014). The assumption is that where the distribution of *p*‐values below .05 are left‐skewed, this indicates a bias towards results that are only marginally statistically significant relative to those that are clearly below an alpha of .05. This can be interpreted as indicating that *p‐*hacking has biased the overall meta‐analytic effect. In this analysis, an estimate of statistical power is provided, with higher power increasing the likelihood of observing a smaller *p*‐value and a ‘true effect’.

## 3 RESULTS

### 3.1 Study selection and study characteristics

The search resulted in 17 eligible studies (see Figure 1). Sample characteristics of the 17 articles included in the meta‐analysis are provided in Table 1. Fifteen effect sizes were calculated for studies reported on specificity as an index of AMT responses, with nine effect sizes being calculated for overgeneral memories. Seven studies reported both specificity and overgeneral memories as an index of AMT responses (Crane et al., 2007; Gupta & Kar, 2012; Haddad et al., 2014; Jermann et al., 2013; Mackinger et al., 2000; Matsumoto et al., 2022).

**TABLE 1:** Characteristics of studies included in Analyses

Author(s)	Year of publication	Sample size	Age‐ *M* (*SD;* range)	Number of females	Education	Race and ethnicity	Diagnostic tool	Number of past episodes in remitted MDD group	Depression severity measure	Number of cues	Cues valence	Memories reported	Duration for responses (seconds)
Aglan, Williams, Pickles, & Hill	2010	*N*_*case*_ = 46 *N*_*control*_ = 57	*M*_*case*_ = 32.96 (2.52; 25–37) *M*_*control*_ = 31.19 (3.05; 25–37)	*N*_*case*_ = 46 *N*_*control*_ = 57	NR	NR	RECAP or SADS (for child); DSM‐IV for adult	45% had >1 past episodes	NR	15	5 positive, 5 negative, 5 neutral	Overgeneral	30
Barnhofer, Crane, Spinhoven, & Williams	2007	*N*_*case*_ = 16 *N*_*control*_ = 19	*M*_*case*_ = 34.44 (14.75; 12–41) *M*_*control*_ = 29.74 (10.74; 12–41)	*N*_*case l*_ = 10 *N*_*control*_ = 11	NR	NR	SCID for DSM‐IV‐TR	Mean of 5.0 past episodes (*SD* = 6.5)	BDI‐II	24	12 positive, 12 negative	Specific	30
Burnside, Startup, Byatt, Rollinson, & Hill	2004	*N*_*case*_ = 18 *N*_*control*_ = 19	*M*_*case*_ = 38.2 (2.41; 31–41) *M*_*control*_ = 35.8 (3.07; 31–41)	*N*_*case*_ = 22 *N*_*control*_ = 19	NR	NR	SADS‐L	NR	BDI	10	5 positive, 5 negative	Overgeneral	30
Champagne et al.	2016	*N*_*case*_ = 25 *N*_*control*_ = 25	*M*_*case*_ = 13.92 (2.27; 11–18) *M*_*control*_ = 13.96 (1.79; 11–18)	*N*_*case*_ = 14 *N*_*control*_ = 13	NR	80% Caucasian in remitted MDD group 92% Caucasian in control group	K‐SADS‐PL	NR	CDI	10	5 positive, 5 negative	Specific	60
Crane, Barnhofer, & Williams	2007	*N*_*case*_ = 23 *N*_*control*_ = 21	*M*_*case*_ = 36.13 (13.92; 18–62) *M*_*control*_ = 29.48 (10.35; 18–62)	*N*_*case*_ = 14 *N*_*control*_ = 12	NR	NR	SCID for DSM‐IV	Mean of 6.8 past episodes (*SD* = 9.7)	BDI‐II	36	18 positive,18 negative	Both	30
Gupta & Kar	2012	*N*_*case*_ = 10 *N*_*control*_ = 10	*M*_*case*_ = 43 (4.49; 38–53) *M*_*control*_ = 43.6 (5.21; 32–50)	*N*_*case*_ = 5 *N*_*control*_ = 5	Education in years for remitted MDD group, *M =* 15.70; *SD* = 3.33 Education in years for control group, *M =* 18.30; *SD* = 2.11	NR	HDRS	NR	HDRS	15	5 positive, 5 negative, 5 neutral	Specific	60
Haddad, Harmer, & Williams	2014	*N*_*case*_ = 24 *N*_*control*_ = 24	*M*_*case*_ = 26.46 (8.22; 18–54) *M*_*control*_ = 24.58 (4.84; 19–54)	*N*_*case*_ = 24 *N*_*control*_ = 24	NR	NR	SCID for DSM‐IV	Mean of 1.9 past episodes (*SD* = 1)	BDI‐II	40	20 positive, 20 negative	Both	20
Haringsma, Spinhoven, Engels, & van der Leeden	2010	*N*_*case*_ = 63 *N*_*control*_ = 60	*M*_*case*_ = 64.92 (6.84; 55–86) *M*_*control*_ = 64.47 (6.65; 55–86)	*N*_*case*_ = 48 *N*_*control*_ = 47	Remitted MDD group: 23.8% completed primary school; 44.4% had 7–11 years in education; 31.8% had ≥12 years in education Control group: 18.33% completed primary school; 48.33% had 7–11 years in education; 33.33% had ≥12 years in education	NR	MINI	Mean of 1.5 (*SD* = 0.5)	CES‐D	10	5 positive, 5 negative	Specific	60
Jermann et al.	2013	*N*_*case*_ = 36 *N*_*control*_ = 20	*M*_*case*_ = 46.8 (10.5) *M*_*control*_ = 45.9 (8.8)	*N*_*case*_ = 25 *N*_*control*_ = 15	Education in years remitted MDD group, *M =* 15.4; *SD* = 3.4 Education in years for control group, *M =* 14.0; *SD* = 3.4	NR	MADRS	Mean of 4.5 past episodes (*SD* = 2.2)	BDI‐II	12	6 positive, 6 negative	Both	NR
Kuyken & Dalgleish	2011	*N*_*case*_ = 15 *N*_*control*_ = 15	*M*_*case*_ = 16.73 (0.8; 14–18) *M*_*control*_ = 16.07 (1.67; 14–18)	*N*_*case*_ = 13 *N*_*control*_ = 10	Highest level of education for remitted MDD group: 27% primary, 60% lower secondary, 13% higher secondary Highest level of education for control group: 20% primary, 73% lower secondary, 7% higher secondary	NR	SCID for DSM‐IV	Mean of 1.2 past episodes (*SD* = 1.1)	BDI‐II	10	5 positive, 5 negative	Both	30
Mackinger, Pachinger, Leibetseder, & Fartacek	2000	*N*_*case*_ = 21 *N*_*control*_ = 20	*M*_*case*_ = 47.9 (11.4; 21–60) *M*_*control*_ = 46 (11; 21–60)	*N*_*case*_ = 21 *N*_*control*_ = 20	NR	NR	DSM‐III‐R	NR	BDI	12	6 positive,6 negative	Both	60
Matsumoto, Takahasi, & Hallford	2021	*N*_*case*_ = 21 *N*_*control*_ = 21	*M*_*case*_ = 30.14 (4.87) *M*_*control*_ = 29.19 (4.69)	*N*_*case*_ = 11 *N*_*control*_ = 12	Education in years remitted MDD group, *M* = 15.10; *SD* = 1.95 Education in years for control group, *M =* 15.67; *SD* = 1.32	100% Japanese sample	SCID for DSM‐IV‐TR	Mean of 1.6 past episodes (*SD* = 1.3)	BDI‐II	10	5 positive, 5 negative	Both	30
Park, Goodyer, & Teasdale	2002	*N*_*case*_ = 9 *N*_*control*_ = 33	*M*_*case*_ = 14.4 (1.1; 12–17) *M*_*control*_ = 14.6 (1.3; 12–17)	*N*_*case*_ = 6 *N*_*control*_ = 21	NR	NR	K‐SADS‐PL	NR	MFQ	12	6 positive, 6 negative	Overgeneral	60
Spinhoven, et al.	2006	*N*_*case*_ = 122 *N*_*control*_ = 37	*M*_*case*_ = 44.7 (9) *M*_*control*_ = 44.2 (13.3)	*N*_*case*_ = 92 *N*_*control*_ = 29	Education level for remitted MDD group: 13.5% low, 35.1% medium, 51.4% high Education level for control group: 29.3% low, 33.6% medium, 37.1% high	NR	SCID for DSM‐IV	Mean of 4.1 past episodes was (*SD* = 1.5)	BDI	10	5 positive, 5 negative	Specific	60
Sumner et al.	2014	*N*_*case*_ = 164 *N*_*control*_ = 275	*M*_*case*_ = 22.5 (0.9; 20–25) *M*_*control*_ = 22.4 (0.9; 20–25)	*N*_*case*_ = 121 *N*_*control*_ = 179	NR	61% Caucasian in remitted MDD group 58% Caucasian in control group	SCID for DSM‐IV	NR	/	12	6 positive, 6 negative	Specific	30
Wessel et al.	2001	*N*_*case*_ = 20 *N*_*control*_ = 24	*M*_*case*_ = 37.5 (8.5; 20–58) *M*_*control*_ = 35.1 (10.4; 20–58)	*N*_*experimental*_ = 13 *N*_*control*_ = 12	Education level for remitted MDD group, *M =* 7.1; *SD* = 2.1 Education level for control group, *M =* 7.2; *SD* = 2.3 (Education level ranged from 1 = *no education* to 11 = *university degree*)	NR	DSM‐III‐R	NR	SDS	10	5 positive, 5 negative	Specific	120
Wessel, Postma, Huntjens, & Crane	2013	*N*_*case*_ = 28 *N*_*control*_ = 33	*M*_*case*_ = 38.71 (7.23; 24–64) *M*_*control*_ = 40 (7.7; 24–64)	*N*_*case*_ = 28 *N*_*control*_ = 33	Education level for remitted MDD group, *M =* 4.5; *SD* = 1.55 Education level for control group, *M =* 5.24; *SD* = 1.25 (Education level ranged from 1 = *elementary school* to 7 = *university degree*)	NR	SCID‐I	NR	BDI‐II	10	5 positive, 5 negative	Specific	30
Williams, Barnhofer, Crane, & Beck	2005	*N*_*case*_ = 34 *N*_*control*_ = 22	*M*_*case*_ = 43.21 (10.70; 18–65) *M*_*control*_ = 49.6 (7.3; 18–65)	*N*_*case*_ = 23 *N*_*control*_ = 13	NR	NR	SCID for DSM‐IV	Mean of 2.7 past episodes (*SD* = 2.1)	BDI‐II	18	6 positive, 6 negative, 6 neutral	Specific	30

All nine studies that reported overgeneral memories in response to the AMT presented number or proportion of categoric memories as an index of overgeneral memory, while only two studies reported the remaining possible overgeneral responses (i.e. extended memories or semantic associates [words that might be thematically related, but not representing a memory per se, e.g. I like flowers]). Given this, as well as previous evidence that differences between people with and without depression may be accounted for by categoric‐type and not extended‐type general memories (Mark et al., 1992), categoric memories were used as the index of overgeneral memory in all analyses.

The sample sizes ranged from nine to 275 participants (range 9–164 for remitted depression; range 10–275 controls). The studies in this review were published between 2000 and 2016, with the exception of one preprint published in 2021. The mean age of participants was 20.9 years (range 11–86 years), with case and control samples across all studies having approximately equal age means. The average proportion of women in the studies was 78.18% (remitted depression group = 82.7%, control = 73.7%). All studies used interviews to assess for depression diagnoses or rule out history of depression, and all but two used structured, standardized diagnostic interview tools.

There were several different measures of depression severity used across samples; however, the most frequently used was the Beck Depression Inventory II (BDI‐II; *n* = 8; 44.4%; Beck et al., 1996). Ten studies provided information about the number of past episodes in the remitted depression group; however, means and standard deviations could only be extracted or calculated from nine. Barnhofer et al. (2007) provided median and range values that were converted to means and standard deviations using the Box–Cox method (McGrath et al., 2020). Haddad et al. (2014) provided the mean and range, the latter of which was used to estimate the standard deviation (Hozo et al., 2005). For Haringsma et al. (2010), the mean and standard deviation were estimated using tabled data indicating that 26 participants had experienced only one previous episode and the remaining 37 participants, who were reported as having two or more previous episodes, were conservatively coded as having two episodes. For Spinhoven et al. (2006), the mean and standard deviation could be estimated from the data provided, although participants that were reported as having six or more previous episodes were conservatively coded as experiencing six episodes.

As required, all studies measured memory specificity or overgenerality using the AMT (Williams & Broadbent, 1986). Furthermore, all studies asked for responses verbally, apart from one study that deviated from this format and required a written response (Wessel et al., 2001). The number of cues provided in order to elicit specific memories in the AMT varied across the studies (range = 10–40). Positive and negative valence was the most commonly reported valences. Too few studies reported neutral valence to assess these responses to these cues separately. Half of the studies allocated a 30‐s response time for AMT cues, while the rest used different response times (range = 20–120 s).

### 3.2 Risk of bias in studies

All studies were found to have at least some concern regarding risk of bias (see the Supporting Information for the full coding of studies). As indicated in Table 2, within each category of possible source of bias, studies were predominantly coded as there being some concern. In most studies, there was no clear concern that data were reported incompletely. No studies were pre‐registered, so the risk of selective reporting could not be ruled out. These findings suggest that studies conducted in this area are likely to have one, if not several, possible sources of bias and should be interpreted accordingly and in the context of indicators of publication bias.

**TABLE 2:** Proportion of studies in categories of potential risk of bias

Random sequence generation																	
Allocation concealment																	
Blinding of participants and personnel																	
Blinding of outcome assessment																	
Incomplete outcome data																	
Selective reporting																	

*Note*: Green = low risk of bias, yellow = some concern, red = High risk of bias.

### 3.3 Results of syntheses: Specific memory retrieval

**FIGURE 2:**
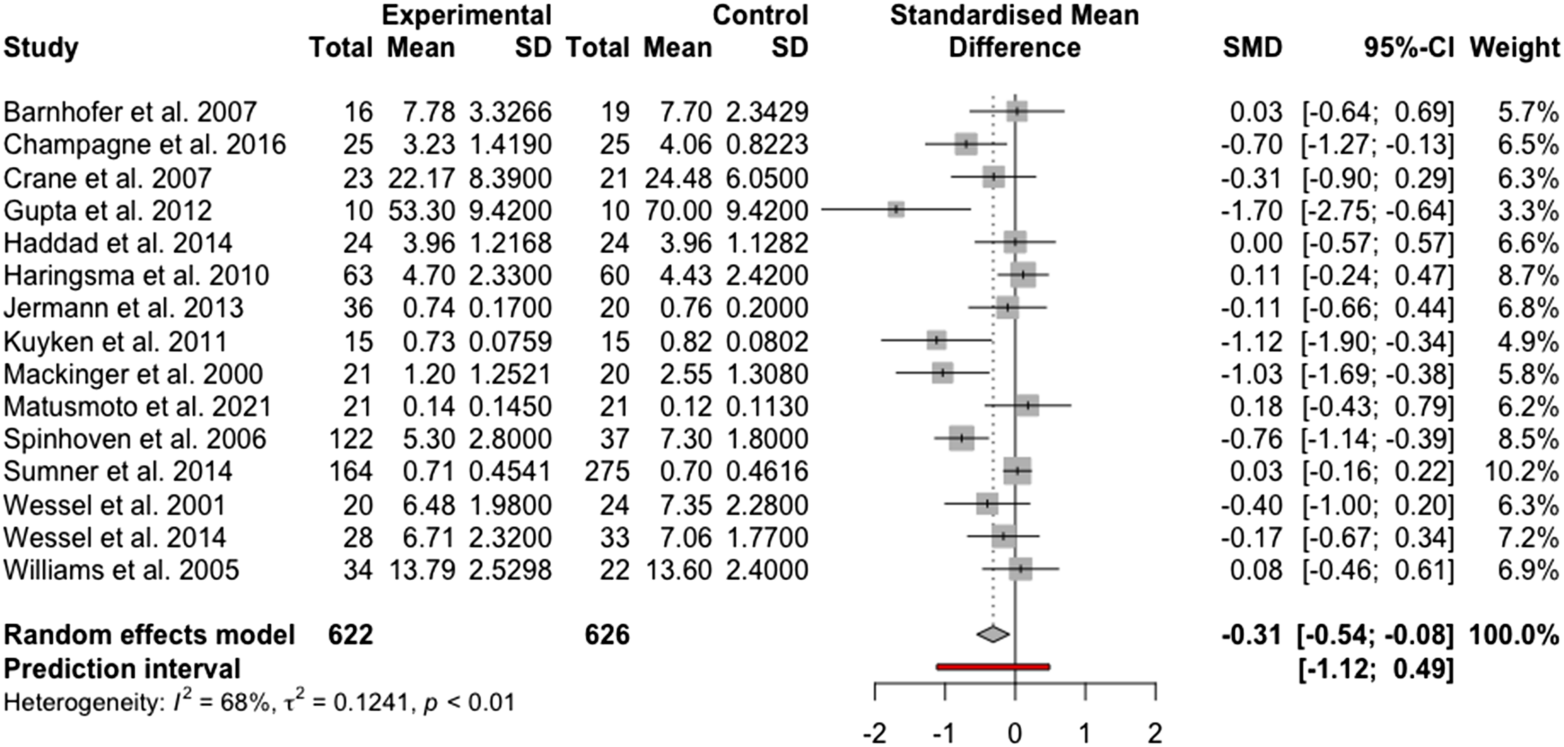
Forest plot of effect sizes for studies assessing specific memories.

There was a small to moderate negative pooled effect for comparisons of specific memories between people with remitted depression compared and people who had never been depressed (*k* = 15; *g* = −0.314, 95% CI [−0.543; −0.085], *z* = −2.69, *p* = .007), suggesting that people in remission recalled fewer specific memories. Regarding heterogeneity, there was evidence of unexplained variance between the study effect sizes (*Q*[14] = 43.41, *p* < .001; *I* ^*2*^ = 67.7%, *τ* ^2^ = .12), indicating a basis for test of moderators that might explain this variance. See Figure 2 for a forest plot of effect sizes, including a prediction interval. The prediction interval indicated that the effect size could be as low as −1.12 in some populations in future studies and as high as +0.54 in others. Moreover, there are also some populations where the effect size could be zero. Outlier analysis indicated there was one outlying effect size (Gupta & Kar, 2012; SMD = −1.69). On omitting this outlier and rerunning the analysis, the results were not substantively changed, and still showed a significant, small to moderate‐sized effect (*k* = 14; *g* = −0.262, 95% CI [−0.477; −0.046], *z* = −2.38, *p* = .017). There was also still evidence for variance between the study effects (*Q*[14] = 35.42, *p* < .001; *I* ^*2*^ = 63.4%, *τ* ^2^ = .09).

#### 3.3.1 Valence effects

On meta‐analysing group differences for studies that examined positively valenced cues separately, the results indicated a significant difference between the remitted depression and never depressed groups, with a small to moderate‐sized effect (*k* = 10; *g* = −0.366, 95% CI [−0.676; −0.056], *z* = −2.31, *p* = .020), and evidence for heterogeneity (*Q*[9] = 40.85, *p* < .001; *I* ^*2*^ = 78%, *τ* ^2^ = .17). One outlier was found (Kuyken & Dalgleish, 2011), and when removed, the effect was largely similar and marginally above the threshold for statistical significance, (*k* = 9; *g* = −0.250, 95% CI[−0.517; 0.016], *z* = −1.84, *p* = .065), with evidence for unexplained variance between the study effect sizes (*Q*[8] = 26.20, *p* = .001; *I* ^*2*^ = 69.5%, *τ* ^2^ = .10). Similarly, for studies that examined negatively valenced cues separately, there was a significant difference between the remitted depression and never depressed, also with a small to moderate‐sized effect (*k* = 10; *g* = −0.324, 95% CI [−0.603; −0.046], *z* = −2.29, *p* = .022) and evidence for heterogeneity (*Q*[9] = 32.81, *p* < .001; *I* ^*2*^ = 72.6%, *τ* ^2^ = .13). One outlier was found (Mackinger et al., 2000), and when removed, the effect was again largely similar and marginally above the threshold for statistical significance (*k* = 9; *g* = −0.222, 95% CI [−0.464; 0.019], *z* = −1.80, *p* = .071), with evidence for unexplained variance between the study effect sizes (*Q*[8] = 26.89, *p* = .007; *I* ^*2*^ = 61.7%, *τ* ^2^ = .07). Given that the effects for positive and negatively valenced cues largely overlapped in their confidence intervals, valence did not appear to be a factor in group difference in specific memories.

#### 3.3.2 Moderator analyses

The differences between the remitted depression and never depressed groups in specific memories were not predicted by differences between the groups in depression symptom severity, *QM* (*df* = 1) = 0.2, *p* = .589, *b* = 0.123, *SE* = 0.214, 95% CI [−0.297; 0.544], *z* = 0.57, *p* = .565, nor by age differences between the groups, *QM* (*df* = 1) = 0.1, *p* = 0.657, *b* = 0.004, *SE* = 0.009, 95% CI [−0.014; 0.022], *z* = 0.43, *p* = .663, proportion of women, *QM* (*df* = 1) = 0.5, *p* = .475, *b* = 0.422, *SE* = 0.591, 95% CI [−0.737; 1.581], *z* = 0.71, *p* = .475, number of cues used for retrieval, *QM* (*df* = 1) = 0.6, *p* = 0.418, *b* = −0.010, *SE* = 0.012, 95% CI [−0.014; 0.035], *z* = 0.81, *p* = .415, retrieval duration, *QM* (*df* = 1) = 2.2, *p* = .136, *b* = 0.038, *SE* = 0.004, 95% CI [−0.015; 0.002], *z* = −1.50, *p* = .132, study age, *QM* (*df* = 1) = 0.1, *p* = .657, *b* = −0.010, *SE* = 0.019, 95% CI [−0.0002; 0.077], *z* = 1.95, *p* = .051, or sample size, *QM* (*df* = 1) = 1.0, *p* = .307, *b* = 0.001, *SE* = 0.001, 95% CI [−0.001; 0.003], *z* = 1.02, *p* = .307. In assessing the number of past episodes of depression as a moderator, one study on adolescents (Kuyken & Dalgleish, 2011) was excluded given this was the only study in this subsample composed of adolescents and that having fewer past depressive episodes was likely to be a function of being substantially younger relative to the remaining samples, which were mostly young to middle‐aged adults. The results from the remaining studies (*k* = 8) indicated no clear evidence for a moderating effect, *b* = −0.099, *SE* = 0.070, 95% CI [−0.237; 0.037], *z* = −1.42, *p* = 0.155. The results did not substantially differ when the adolescent study was included.

#### 3.3.3 Publication bias

**FIGURE 3:**
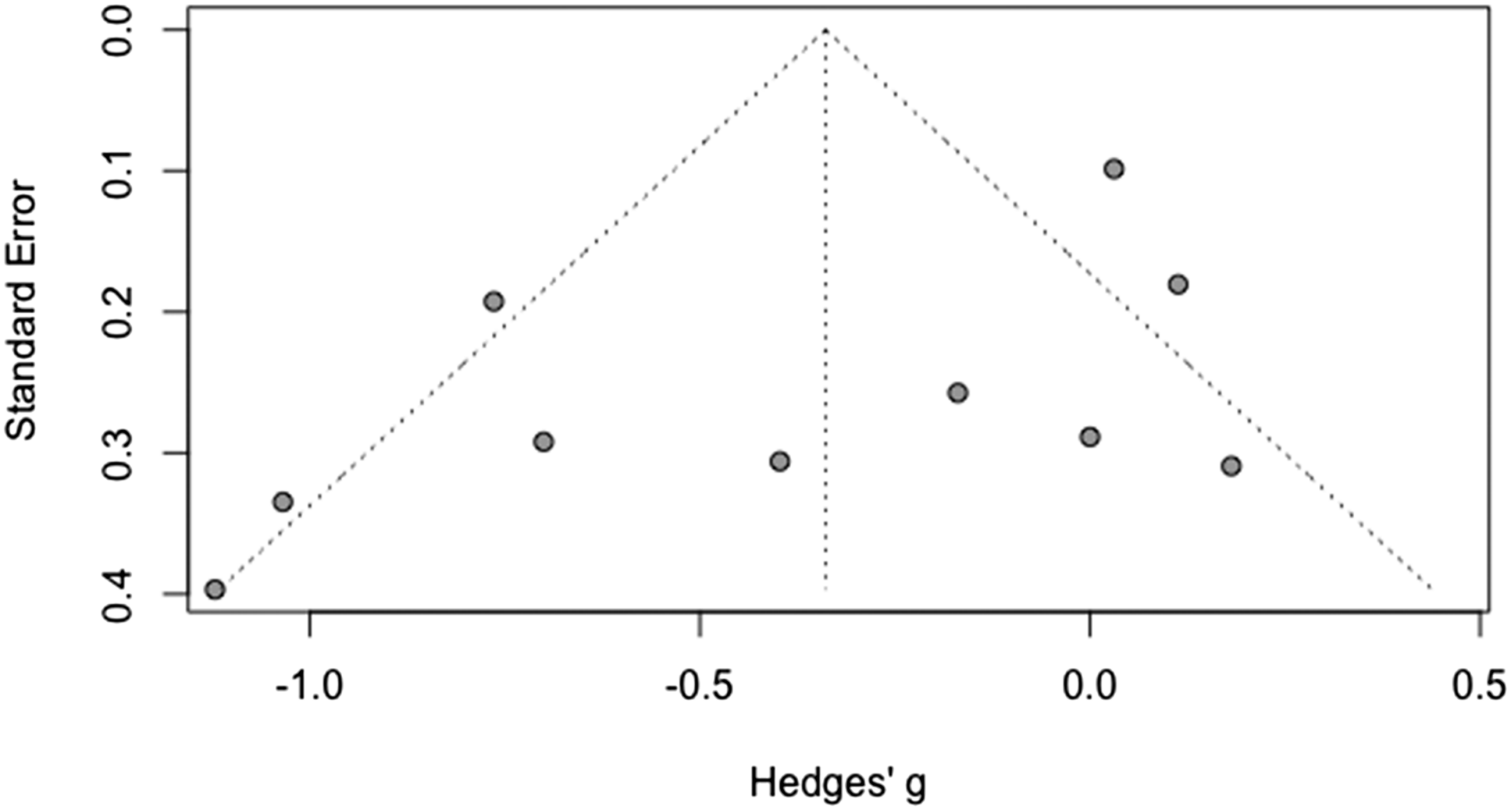
Funnel plot of studies assessing specific memories.

Figure 3 shows a funnel plot of the full sample of studies assessing specificity. The results of Eggers' test was suggestive of funnel plot asymmetry, intercept = −2.15, 95% CI [−3.95; −2.33], *p* = 0.035. The trim‐and‐fill procedure suggested adding two studies to the right side of the mean, which produced an attenuated effect size that had overlapping confidence intervals with the original estimate of group differences (*k* = 17; *g* = −0.212, 95% CI [−0.487; 0.161], *z* = −1.52, *p* = 128). The results of the *P*‐curve analysis showed that there was adequate power to detect an effect, 80% (95% CI 41.5–95.9%), and that there was evidence of a ‘true’ effect in the overall findings. Taken together, these analyses indicate that observed group differences in specificity were not the product of ‘*p*‐hacking’, but there may be some publication bias that attenuates the observed effect.

### 3.4 Results of syntheses: Categoric memory retrieval

**FIGURE 4:**
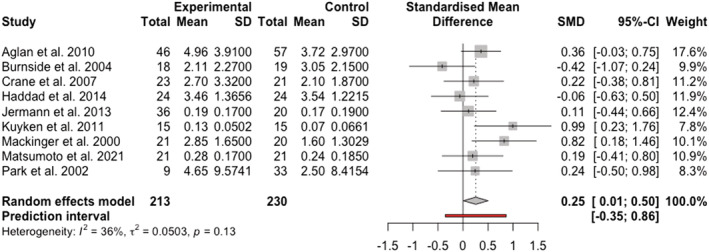
Forest plot of effect sizes for studies assessing categoric memories.

There was a small to moderate pooled effect for comparisons of categoric memories between people with remitted depression compared to people who had never been depressed (*k* = 9; *g* = 0.254, 95% CI [0.007; 0.501], *z* = 2.02, *p* = .043), suggesting that people in remission recalled more categoric people without any history of depression. A test for heterogeneity indicated there was not clear evidence for unexplained variance between the study effect sizes (*Q*[8] = 12.49, *p* = .134; *I* ^*2*^ = 36%, *τ* ^2^ = .05). See Figure 4 for a forest plot of effect sizes, including a prediction interval. Consistent with specific memory analysis, the prediction interval crossed zero and indicated that the effect size could be as low as −0.35, as high as +0.86 or zero in some population's in future studies. No outliers were detected.

#### 3.4.1 Valence effects

On meta‐analysing group differences for studies that examined positively valenced cues separately, the results indicated a significant difference between the remitted depression and never depressed groups, with a small to moderate‐sized effect (*k* = 7; *g* = 0.339, 95% CI [0.058; 0.619], *z* = 2.37, *p* = .017), with no clear evidence of heterogeneity (*Q*[6] = 9.17, *p* = .164; *I* ^*2*^ = 34.6%, *τ* ^2^ = .04). No outliers were detected. For studies that examined negatively valenced cues separately, there was a small to moderate but non‐significant difference between the remitted depression and never depressed groups (*k* = 7; *g* = 0.278, 95% CI [−0.182; 0.740], *z* = 1.19, *p* = .235) and evidence for heterogeneity (*Q*[6] = 23.99, *p* < .001; *I* ^*2*^ = 75%, *τ* ^2^ = .28). No outliers were found. In summary, a significant effect was found for positively valenced cues, but not for negatively valenced cues. However, given the similarity in their effect size and confidence intervals that predominantly overlapped, this did not provide clear evidence that cue valence was a factor in group differences in categoric memories.

#### 3.4.2 Moderator analyses

The difference between the remitted depression and never depressed groups in categoric memories (across cue valences) was not predicted by differences between the groups in depression symptom severity, *QM* (*df* = 1) = 2.8, *p* = .092, *b* = −.400, *SE* = 0.245, 95% CI [−0.881; 0.081], *z* = −1.62, *p* = .092, nor age differences between the groups, *QM* (*df* = 1) = .09, *p* = .752, *b* = −0.004, *SE* = 0.12, 95% CI [−0.029; 0.021], *z* = −0.31, *p* = .753, proportion of women, *QM* (*df* = 1) = 0.02, *p* = .875, *b* = −0.111, *SE* = 0.715, 95% CI [−1.51; 1.29], *z* = −0.15, *p* = .875, number of cues used for retrieval, *QM* (*df* = 1) = 0.5, *p* = .442, *b* = −0.008, *SE* = 0.011, 95% CI [−0.031; 0.014], *z* = −0.74, *p* = .454, retrieval duration, *QM* (*df* = 1) = 1.7, *p* = .190, *b* = 0.012, *SE* = 0.010, 95% CI[−0.007; 0.032], *z* = 1.23, *p* = .216, study age, *QM* (*df* = 1) = 0.2, *p* = .625, *b* = −0.010, *SE* = 0.021, 95% CI [−0.052; 0.031], *z* = −0.48, *p* = .625, or sample size, *QM* (*df* = 1) = 0.0009, *p* = .976, *b* = 0.002, *SE* = 0.006, 95% CI [−0.011; 0.011], *z* = 0.29, *p* = .976. Again, when assessing past episodes of depression, a study with adolescents was excluded (Kuyken & Dalgleish, 2011). This left few studies (*k* = 4), among which there was no clear evidence for a moderating effect, *b* = 0.028, *SE* = 0.071, 95% CI [−0.111; 0.167], *z* = 0.039, *p* = .693. Notably, this analysis had very low statistical power.

#### 3.4.3 Publication bias

**FIGURE 5:**
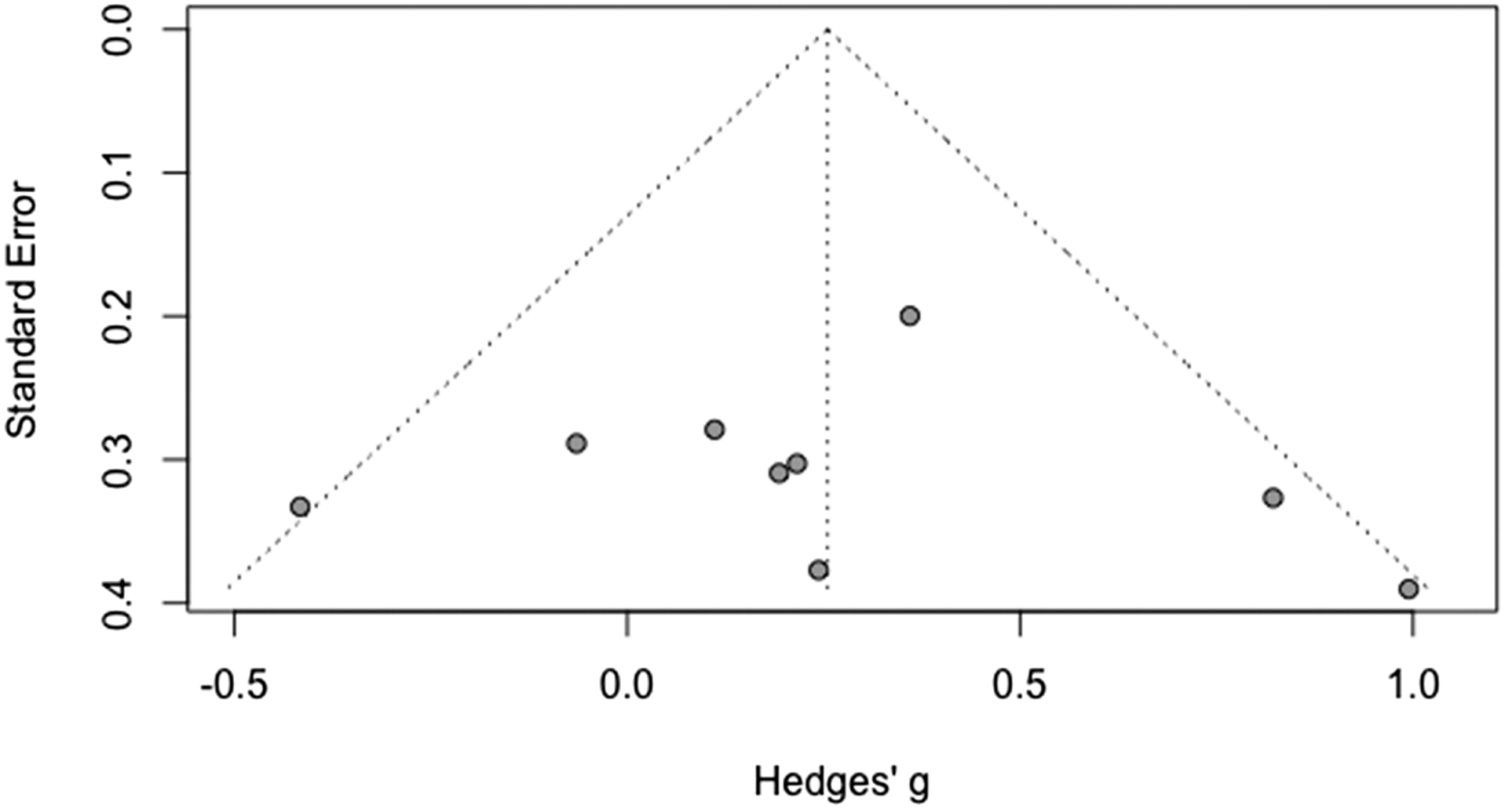
Funnel plot of studies assessing categoric memories.

Figure 5 shows a funnel plot of the full sample of studies assessing categoric memories. The results of Eggers' test did not indicate the presence of funnel plot asymmetry, intercept = 0.51, 95% CI [−3.84; 4.87], *p* = .823, and therefore did not support the presence of publication bias. The trim‐and‐fill procedure did not suggest adding any studies. Although statistical power was low for Eggers test (*k* < 10), the results were highly non‐significant and not suggestive of a false‐negative result for bias. As there were two or less *p*‐values less than .05, *p*‐curve analysis could not be conducted.

## 4 DISCUSSION

This study systematically sampled and reviewed extant literature to examine whether or not, and to what extent, reduced specificity or overgenerality in autobiographical memory retrieval persists following the remission of depression. To do this, studies that compared groups of people with a history of clinical depression and no current depression to people with no historical or current clinical depression were included. The results of the meta‐analyses indicated that small to moderate deficits in the recall of specific, event‐level personal memories are apparent among people who have remitted from clinical depression. In combination with other existing meta‐analyses showing that the presence of depression diagnoses is also associated with reduced specificity (Liu et al., 2013; Williams et al., 2007), our findings therefore provide some evidence for a cognitive deficit that persists beyond episodes of major depression. This is significant, as evidence shows that difficulty in retrieving event‐level, specific personal memories is prognostic of future depressive symptoms (Hallford, Rusanov, et al., 2021). Therefore, our study provides further evidence that reduced memory specificity/overgeneral memory may be a risk factor for future episodes of depression in those that are in remission. The between group effect sizes were smaller than those typically found in studies and meta‐analyses that compare people with active clinical depression to those who are not depressed (Liu et al., 2013; Williams et al., 2007) This suggests that there may be other processes associated with being depressed that make the remembering of specific, event‐level memories harder.

There was not strong evidence that memory specificity varied based on the valence of cues. This indicates that people with a history of depression recall fewer memories of specific personal events that occurred in particular places and times in their lives regardless of whether this is prompted by a positive or negatively valenced word. Although cue valence does not correspond to the emotional valence of memory in a 1:1 manner, evidence indicates that the majority of responses on the AMT will have an emotional valence consistent with the cue word (Young et al., 2012).

Interestingly, the observed impairments in autobiographical memory were not explained by differences in current depressive symptoms between the groups. Therefore, it was not residual depressive symptoms or a generally higher baseline level of subclinical depressive symptoms in those with remitted depression that accounted for these differences. The substantial heterogeneity in the analyses of specific and categoric memories was not explained by any of the other moderators either. This heterogeneity is associated with wide confidence intervals around the point estimates, and therefore, although there does appear to be observable group differences, there are unknown factors that predict when these differences are likely to be smaller or larger. The risk of bias assessment provided some evidence of bias for studies reporting specific memories, suggesting an attenuated effect size. However, it is important to note that the heterogeneity between studies may limited the interpretability of this finding. There was no clear evidence of *p*‐hacking; however, the risk of bias within studies indicated possible causes for concern across all studies.

### 4.1 Limitations and future directions

There were few studies that examined older aged adults, thereby constraining the analysis of age effects primarily to the range of adolescence to middle‐aged adults. Future research in remitted depression in older adult samples is needed. There was a lack of reporting of co‐occurring disorders or related clinical symptoms, and therefore, whether there was an additive effect of further pathology on group differences is unclear. There were few studies that reported on number of previous episodes and no clear evidence that this moderated the observed effects. This may be an important factor to consider in future research though, given that the severity of other deficits in cognitive functioning that persist after the remission of depression does appear to be predicted by higher numbers of previous episodes (Semkovska et al., 2019) Further, while this review can confirm deficits in specific memories, and a tendency to instead recall a greater number of categoric memories, in remitted depression, it cannot ascertain if this was a precursor to people's initial episode, or whether this occurred as a result of depression and then continued as a risk factor for future episodes, that is, the ‘scar’ hypothesis for depression (Lewinsohn et al., 1981). Prospective studies are needed to assess if people who experience clinical depression already have relatively lower pre‐morbid memory specificity than those who do not experience clinical depression or whether these deficits emerge and persist as a result of clinical depression.

The current study focused solely on the AMT and on indices of memory specificity/overgenerality in remitted depression. While this was the pre‐stated focus of the study, and the AMT is the most commonly used task within the memory specificity literature (Barry, Hallford, & Takano, 2021), it does mean that only a limited picture of autobiographical memory in remitted depression is presented. There are other, associated characteristics of autobiographical memory that warrant study in remitted depression in the future, such as the amount of episodic or semantic details (Hallford, Barry, et al., 2021) and use of mental imagery (Mansell & Lam, 2004). It is important to note that although evidence was found for significant mean group differences using the available data, there were studies with no effect and studies with effects in the opposite direction (albeit not significant). Further, the prediction intervals suggested that it is possible that future studies will find no differences or differences of the reverse direction in future studies. It is unclear why this might be the case, given that there were no apparent moderators of group differences in the available data. Therefore, there was no clear signal as to what factors might lead to no effect or an opposite effect. Given the effects that were observed were not large, it is possible that some this variation is due to sampling error, and most studies had small samples. Nonetheless, future studies should continue to include a raft of potential moderating variables, not limited to those here. Further studies in remitted depression also appear to be required to establish the robustness of the finding.

Another limitation of this study is that it did not directly compare differences in specificity between people with remitted depression and current depression. Indeed, the differences observed here between those with remitted depression and without any history of depression are notably smaller than those observed between current depression and no history of depression (Barry, Hallford, & Takano, 2021). How can this be interpreted? Processes associated with reduced specificity/overgenerality and depression, such as rumination or executive functioning deficits, do not necessarily completely resolve with depressive symptoms. Indeed, higher rumination (Visted et al., 2018) and impaired executive functioning (Rock et al., 2014) do appear to be characteristics of remitted depression suggesting they persist once a person no longer meets criteria for major depression and may disrupt access to specific autobiographical memories. Another possibility is that it represents a continuation of the learned response of functional avoidance of specific memories. Williams et al. (2006) proposed that people may avoid retrieving negatively valenced specific memories as a means of regulating their emotion and that over time this tendency may become over compensatory and generalize to the tendency to avoid all specific memories, in the vein of ‘better safe than sorry’ (van den Bergh et al., 2021). Therefore, although depressive symptoms may remit, the tendency to avoid specific memories may persist.

With respect to future research, this study suggests that memory specificity is a suitable target for intervention not only in the treatment of active depression (Barry, Hallford, Hitchcock, et al., 2021) but potentially also in the reduction of relapse. Fortunately, there is now robust evidence that memory specificity is a modifiable cognitive variable, both in clinical and non‐clinical samples (Barry, Sze, & Raes, 2019). Indeed, recent research has indicated that improvement in memory specificity can be achieved through face‐to‐face (Barry, Sze, & Raes, 2019) and automated, online methods (Hallford, Austin, et al., 2021).

As reduced specificity/overgenerality of personal memories appears to be transdiagnostic in nature (Barry, Hallford, & Takano, 2021), it may well persist beyond remission in other mental disorders. Through our search of the literature, there was little evidence of studies that investigated this outside of depression, and therefore, this represents an avenue for future research. Another consideration for future research is that future thinking specificity is also markedly diminished in those with clinical depression (Hallford, Austin, et al., 2018), and whether or not these difficulties also persist beyond remission has yet to be tested.

### 4.2 Conclusion

In conclusion, this review provides evidence that people who are in remission from clinical depression have difficulties retrieving specific autobiographical memories and instead recall more categoric memories, relative to those that have no history of depression. Although these effects are only small to moderate in size, it is a factor that is known to predict future depressive symptoms. Knowledge that this difficulty persists even when people are otherwise free from depression may inform programmes designed to reduce depressive relapse and the immense personal and societal burden of this illness.

## CONFLICT OF INTEREST

No conflicts to declare.

## Supporting information


**Data S1.** Supporting Information

**Data S2.** Supporting Information

## Data Availability

The data and scripts used for analysis in this study are open access and available at https://osf.io/bfcyj/ (noted below in manuscript).
