# Supplementary material for: Reduced specificity and increased overgenerality of autobiographical memory persist as cognitive vulnerabilities in remitted major depression: A meta‐analysis
Source: Clin Psychol Psychother. 2022 Oct 4;29(5):1515–29. doi: 10.1002/cpp.2786 (PMC9828164; doi:10.1002/cpp.2786)
Supplement: Supplementary file 2 — Data S2. Supporting Information [file CPP-29-1515-s002.docx]

**Post traumatic stress**

(Post traumatic stress AND “overgeneral memory”) OR (Post traumatic stress AND “memory specificity”) OR (Post traumatic stress AND "memory specificity" AND ruminat*) OR (Post traumatic stress AND "memory specificity" AND brooding) OR (Post traumatic stress AND "memory specificity" AND reflection) OR (Post traumatic stress AND "memory specificity" AND "executive function") OR (Post traumatic stress AND "memory specificity" AND "executive control") OR (Post traumatic stress AND "memory specificity" AND avoidance) OR (Post traumatic stress AND "memory specificity" AND "verbal fluency") OR (Post traumatic stress AND "memory specificity" AND "semantic fluency") OR (Post traumatic stress AND "memory specificity" AND "inhibitory control") OR (Post traumatic stress AND "memory specificity" AND updating) OR (Post traumatic stress AND "memory specificity" AND "repetitive thinking") OR (Post traumatic stress AND "memory specificity" AND worry) OR (Post traumatic stress AND "memory specificity" AND "problem solving") OR (Post traumatic stress AND "memory specificity" AND "future thinking") OR (Post traumatic stress AND "overgeneral memory" AND ruminat*) OR (Post traumatic stress AND "overgeneral memory" AND brooding) OR (Post traumatic stress AND "overgeneral memory" AND reflection) OR (Post traumatic stress AND "overgeneral memory" AND "executive function") OR (Post traumatic stress AND "overgeneral memory" AND "executive control") OR (Post traumatic stress AND "overgeneral memory" AND avoidance) OR (Post traumatic stress AND "overgeneral memory" AND "verbal fluency") OR (Post traumatic stress AND "overgeneral memory" AND "semantic fluency") OR (Post traumatic stress AND "overgeneral memory" AND "inhibitory control") OR (Post traumatic stress AND "overgeneral memory" AND updating) OR (Post traumatic stress AND "overgeneral memory" AND "repetitive thinking") OR (Post traumatic stress AND "overgeneral memory" AND worry) OR (Post traumatic stress AND "overgeneral memory" AND "problem solving") OR (Post traumatic stress AND "overgeneral memory" AND "future thinking")

**Depression**

(Depression AND “overgeneral memory” ) OR (Depression AND “memory specificity”) OR (Depression AND "memory specificity" AND ruminat*) OR (Depression AND "memory specificity" AND brooding) OR (Depression AND "memory specificity" AND reflection) OR (Depression AND "memory specificity" AND "executive function") OR (Depression AND "memory specificity" AND "executive control") OR (Depression AND "memory specificity" AND avoidance) OR (Depression AND "memory specificity" AND "verbal fluency") OR (Depression AND "memory specificity" AND "semantic fluency") OR (Depression AND "memory specificity" AND "inhibitory control") OR (Depression AND "memory specificity" AND updating) OR (Depression AND "memory specificity" AND "repetitive thinking") OR (Depression AND "memory specificity" AND worry) OR (Depression AND "memory specificity" AND "problem solving") OR (Depression AND "memory specificity" AND "future thinking") OR (Depression AND "overgeneral memory" AND ruminat*) OR (Depression AND "overgeneral memory" AND brooding) OR (Depression AND "overgeneral memory" AND reflection) OR (Depression AND "overgeneral memory" AND "executive function") OR (Depression AND "overgeneral memory" AND "executive control") OR (Depression AND "overgeneral memory" AND avoidance) OR (Depression AND "overgeneral memory" AND "verbal fluency") OR (Depression AND "overgeneral memory" AND "semantic fluency") OR (Depression AND "overgeneral memory" AND "inhibitory control") OR (Depression AND "overgeneral memory" AND updating) OR (Depression AND "overgeneral memory" AND "repetitive thinking") OR (Depression AND "overgeneral memory" AND worry) OR (Depression AND "overgeneral memory" AND "problem solving") OR (Depression AND "overgeneral memory" AND "future thinking")

**Bipolar**

(Bipolar AND "memory specificity") OR (Bipolar AND "overgeneral memory") OR (Bipolar AND "memory specificity" AND ruminat*) OR (Bipolar AND "memory specificity" AND brooding) OR (Bipolar AND "memory specificity" AND reflection) OR (Bipolar AND "memory specificity" AND "executive function") OR (Bipolar AND "memory specificity" AND "executive control") OR (Bipolar AND "memory specificity" AND avoidance) OR (Bipolar AND "memory specificity" AND "verbal fluency") OR (Bipolar AND "memory specificity" AND "semantic fluency") OR (Bipolar AND "memory specificity" AND "inhibitory control") OR (Bipolar AND "memory specificity" AND updating) OR (Bipolar AND "memory specificity" AND "repetitive thinking") OR (Bipolar AND "memory specificity" AND worry) OR (Bipolar AND "memory specificity" AND "problem solving") OR (Bipolar AND "memory specificity" AND "future thinking") OR (Bipolar AND "overgeneral memory" AND ruminat*) OR (Bipolar AND "overgeneral memory" AND brooding) OR (Bipolar AND "overgeneral memory" AND reflection) OR (Bipolar AND "overgeneral memory" AND "executive function") OR (Bipolar AND "overgeneral memory" AND "executive control") OR (Bipolar AND "overgeneral memory" AND avoidance) OR (Bipolar AND "overgeneral memory" AND "verbal fluency") OR (Bipolar AND "overgeneral memory" AND "semantic fluency") OR (Bipolar AND "overgeneral memory" AND "inhibitory control") OR (Bipolar AND "overgeneral memory" AND updating) OR (Bipolar AND "overgeneral memory" AND "repetitive thinking") OR (Bipolar AND "overgeneral memory" AND worry) OR (Bipolar AND "overgeneral memory" AND "problem solving") OR (Bipolar AND "overgeneral memory" AND "future thinking")

**Schizophrenia**

(Schizophrenia AND “overgeneral memory” ) OR (Schizophrenia AND “memory specificity”) OR (Schizophrenia AND "memory specificity" AND ruminat*) OR (Schizophrenia AND "memory specificity" AND brooding) OR (Schizophrenia AND "memory specificity" AND reflection) OR (Schizophrenia AND "memory specificity" AND "executive function") OR (Schizophrenia AND "memory specificity" AND "executive control") OR (Schizophrenia AND "memory specificity" AND avoidance) OR (Schizophrenia AND "memory specificity" AND "verbal fluency") OR (Schizophrenia AND "memory specificity" AND "semantic fluency") OR (Schizophrenia AND "memory specificity" AND "inhibitory control") OR (Schizophrenia AND "memory specificity" AND updating) OR (Schizophrenia AND "memory specificity" AND "repetitive thinking") OR (Schizophrenia AND "memory specificity" AND worry) OR (Schizophrenia AND "memory specificity" AND "problem solving") OR (Schizophrenia AND "memory specificity" AND "future thinking") OR (Schizophrenia AND "overgeneral memory" AND ruminat*) OR (Schizophrenia AND "overgeneral memory" AND brooding) OR (Schizophrenia AND "overgeneral memory" AND reflection) OR (Schizophrenia AND "overgeneral memory" AND "executive function") OR (Schizophrenia AND "overgeneral memory" AND "executive control") OR (Schizophrenia AND "overgeneral memory" AND avoidance) OR (Schizophrenia AND "overgeneral memory" AND "verbal fluency") OR (Schizophrenia AND "overgeneral memory" AND "semantic fluency") OR (Schizophrenia AND "overgeneral memory" AND "inhibitory control") OR (Schizophrenia AND "overgeneral memory" AND updating) OR (Schizophrenia AND "overgeneral memory" AND "repetitive thinking") OR (Schizophrenia AND "overgeneral memory" AND worry) OR (Schizophrenia AND "overgeneral memory" AND "problem solving") OR (Schizophrenia AND "overgeneral memory" AND "future thinking")

**Anxiety disorder**

(Anxiety disorder AND “overgeneral memory” ) OR (Anxiety disorder AND “memory specificity”) OR (Anxiety disorder AND "memory specificity" AND ruminat*) OR (Anxiety disorder AND "memory specificity" AND brooding) OR (Anxiety disorder AND "memory specificity" AND reflection) OR (Anxiety disorder AND "memory specificity" AND "executive function") OR (Anxiety disorder AND "memory specificity" AND "executive control") OR (Anxiety disorder AND "memory specificity" AND avoidance) OR (Anxiety disorder AND "memory specificity" AND "verbal fluency") OR (Anxiety disorder AND "memory specificity" AND "semantic fluency") OR (Anxiety disorder AND "memory specificity" AND "inhibitory control") OR (Anxiety disorder AND "memory specificity" AND updating) OR (Anxiety disorder AND "memory specificity" AND "repetitive thinking") OR (Anxiety disorder AND "memory specificity" AND worry) OR (Anxiety disorder AND "memory specificity" AND "problem solving") OR (Anxiety disorder AND "memory specificity" AND "future thinking") OR (Anxiety disorder AND "overgeneral memory" AND ruminat*) OR (Anxiety disorder AND "overgeneral memory" AND brooding) OR (Anxiety disorder AND "overgeneral memory" AND reflection) OR (Anxiety disorder AND "overgeneral memory" AND "executive function") OR (Anxiety disorder AND "overgeneral memory" AND "executive control") OR (Anxiety disorder AND "overgeneral memory" AND avoidance) OR (Anxiety disorder AND "overgeneral memory" AND "verbal fluency") OR (Anxiety disorder AND "overgeneral memory" AND "semantic fluency") OR (Anxiety disorder AND "overgeneral memory" AND "inhibitory control") OR (Anxiety disorder AND "overgeneral memory" AND updating) OR (Anxiety disorder AND "overgeneral memory" AND "repetitive thinking") OR (Anxiety disorder AND "overgeneral memory" AND worry) OR (Anxiety disorder AND "overgeneral memory" AND "problem solving") OR (Anxiety disorder AND "overgeneral memory" AND "future thinking")

**Panic Disorder**

(Panic Disorder AND "memory specificity") OR (Panic Disorder AND "overgeneral memory") OR (Panic Disorder AND "memory specificity" AND ruminat*) OR (Panic Disorder AND "memory specificity" AND brooding) OR (Panic Disorder AND "memory specificity" AND reflection) OR (Panic Disorder AND "memory specificity" AND "executive function") OR (Panic Disorder AND "memory specificity" AND "executive control") OR (Panic Disorder AND "memory specificity" AND avoidance) OR (Panic Disorder AND "memory specificity" AND "verbal fluency") OR (Panic Disorder AND "memory specificity" AND "semantic fluency") OR (Panic Disorder AND "memory specificity" AND "inhibitory control") OR (Panic Disorder AND "memory specificity" AND updating) OR (Panic Disorder AND "memory specificity" AND "repetitive thinking") OR (Panic Disorder AND "memory specificity" AND worry) OR (Panic Disorder AND "memory specificity" AND "problem solving") OR (Panic Disorder AND "memory specificity" AND "future thinking") OR (Panic Disorder AND "overgeneral memory" AND ruminat*) OR (Panic Disorder AND "overgeneral memory" AND brooding) OR (Panic Disorder AND "overgeneral memory" AND reflection) OR (Panic Disorder AND "overgeneral memory" AND "executive function") OR (Panic Disorder AND "overgeneral memory" AND "executive control") OR (Panic Disorder AND "overgeneral memory" AND avoidance) OR (Panic Disorder AND "overgeneral memory" AND "verbal fluency") OR (Panic Disorder AND "overgeneral memory" AND "semantic fluency") OR (Panic Disorder AND "overgeneral memory" AND "inhibitory control") OR (Panic Disorder AND "overgeneral memory" AND updating) OR (Panic Disorder AND "overgeneral memory" AND "repetitive thinking") OR (Panic Disorder AND "overgeneral memory" AND worry) OR (Panic Disorder AND "overgeneral memory" AND "problem solving") OR (Panic Disorder AND "overgeneral memory" AND "future thinking")

**Social Anxiety Disorder**

(Social Anxiety Disorder AND "memory specificity") OR (Social Anxiety Disorder AND "overgeneral memory") OR (Social Anxiety Disorder AND "memory specificity" AND ruminat*) OR (Social Anxiety Disorder AND "memory specificity" AND brooding) OR (Social Anxiety Disorder AND "memory specificity" AND reflection) OR (Social Anxiety Disorder AND "memory specificity" AND "executive function") OR (Social Anxiety Disorder AND "memory specificity" AND "executive control") OR (Social Anxiety Disorder AND "memory specificity" AND avoidance) OR (Social Anxiety Disorder AND "memory specificity" AND "verbal fluency") OR (Social Anxiety Disorder AND "memory specificity" AND "semantic fluency") OR (Social Anxiety Disorder AND "memory specificity" AND "inhibitory control") OR (Social Anxiety Disorder AND "memory specificity" AND updating) OR (Social Anxiety Disorder AND "memory specificity" AND "repetitive thinking") OR (Social Anxiety Disorder AND "memory specificity" AND worry) OR (Social Anxiety Disorder AND "memory specificity" AND "problem solving") OR (Social Anxiety Disorder AND "memory specificity" AND "future thinking") OR (Social Anxiety Disorder AND "overgeneral memory" AND ruminat*) OR (Social Anxiety Disorder AND "overgeneral memory" AND brooding) OR (Social Anxiety Disorder AND "overgeneral memory" AND reflection) OR (Social Anxiety Disorder AND "overgeneral memory" AND "executive function") OR (Social Anxiety Disorder AND "overgeneral memory" AND "executive control") OR (Social Anxiety Disorder AND "overgeneral memory" AND avoidance) OR (Social Anxiety Disorder AND "overgeneral memory" AND "verbal fluency") OR (Social Anxiety Disorder AND "overgeneral memory" AND "semantic fluency") OR (Social Anxiety Disorder AND "overgeneral memory" AND "inhibitory control") OR (Social Anxiety Disorder AND "overgeneral memory" AND updating) OR (Social Anxiety Disorder AND "overgeneral memory" AND "repetitive thinking") OR (Social Anxiety Disorder AND "overgeneral memory" AND worry) OR (Social Anxiety Disorder AND "overgeneral memory" AND "problem solving") OR (Social Anxiety Disorder AND "overgeneral memory" AND "future thinking")

**General Anxiety Disorder**

(General Anxiety Disorder AND "memory specificity") OR (General Anxiety Disorder AND "overgeneral memory") OR (General Anxiety Disorder AND "memory specificity" AND ruminat*) OR (General Anxiety Disorder AND "memory specificity" AND brooding) OR (General Anxiety Disorder AND "memory specificity" AND reflection) OR (General Anxiety Disorder AND "memory specificity" AND "executive function") OR (General Anxiety Disorder AND "memory specificity" AND "executive control") OR (General Anxiety Disorder AND "memory specificity" AND avoidance) OR (General Anxiety Disorder AND "memory specificity" AND "verbal fluency") OR (General Anxiety Disorder AND "memory specificity" AND "semantic fluency") OR (General Anxiety Disorder AND "memory specificity" AND "inhibitory control") OR (General Anxiety Disorder AND "memory specificity" AND updating) OR (General Anxiety Disorder AND "memory specificity" AND "repetitive thinking") OR (General Anxiety Disorder AND "memory specificity" AND worry) OR (General Anxiety Disorder AND "memory specificity" AND "problem solving") OR (General Anxiety Disorder AND "memory specificity" AND "future thinking") OR (General Anxiety Disorder AND "overgeneral memory" AND ruminat*) OR (General Anxiety Disorder AND "overgeneral memory" AND brooding) OR (General Anxiety Disorder AND "overgeneral memory" AND reflection) OR (General Anxiety Disorder AND "overgeneral memory" AND "executive function") OR (General Anxiety Disorder AND "overgeneral memory" AND "executive control") OR (General Anxiety Disorder AND "overgeneral memory" AND avoidance) OR (General Anxiety Disorder AND "overgeneral memory" AND "verbal fluency") OR (General Anxiety Disorder AND "overgeneral memory" AND "semantic fluency") OR (General Anxiety Disorder AND "overgeneral memory" AND "inhibitory control") OR (General Anxiety Disorder AND "overgeneral memory" AND updating) OR (General Anxiety Disorder AND "overgeneral memory" AND "repetitive thinking") OR (General Anxiety Disorder AND "overgeneral memory" AND worry) OR (General Anxiety Disorder AND "overgeneral memory" AND "problem solving") OR (General Anxiety Disorder AND "overgeneral memory" AND "future thinking")

**Agoraphobia**

(Agoraphobia AND "memory specificity") OR (Agoraphobia AND "overgeneral memory")OR (Agoraphobia AND "memory specificity" AND ruminat*) OR (Agoraphobia AND "memory specificity" AND brooding) OR (Agoraphobia AND "memory specificity" AND reflection) OR (Agoraphobia AND "memory specificity" AND "executive function") OR (Agoraphobia AND "memory specificity" AND "executive control") OR (Agoraphobia AND "memory specificity" AND avoidance) OR (Agoraphobia AND "memory specificity" AND "verbal fluency") OR (Agoraphobia AND "memory specificity" AND "semantic fluency") OR (Agoraphobia AND "memory specificity" AND "inhibitory control") OR (Agoraphobia AND "memory specificity" AND updating) OR (Agoraphobia AND "memory specificity" AND "repetitive thinking") OR (Agoraphobia AND "memory specificity" AND worry) OR (Agoraphobia AND "memory specificity" AND "problem solving") OR (Agoraphobia AND "memory specificity" AND "future thinking") OR (Agoraphobia AND "overgeneral memory" AND ruminat*) OR (Agoraphobia AND "overgeneral memory" AND brooding) OR (Agoraphobia AND "overgeneral memory" AND reflection) OR (Agoraphobia AND "overgeneral memory" AND "executive function") OR (Agoraphobia AND "overgeneral memory" AND "executive control") OR (Agoraphobia AND "overgeneral memory" AND avoidance) OR (Agoraphobia AND "overgeneral memory" AND "verbal fluency") OR (Agoraphobia AND "overgeneral memory" AND "semantic fluency") OR (Agoraphobia AND "overgeneral memory" AND "inhibitory control") OR (Agoraphobia AND "overgeneral memory" AND updating) OR (Agoraphobia AND "overgeneral memory" AND "repetitive thinking") OR (Agoraphobia AND "overgeneral memory" AND worry) OR (Agoraphobia AND "overgeneral memory" AND "problem solving") OR (Agoraphobia AND "overgeneral memory" AND "future thinking")

**Specific phobia**

(Specific phobia AND "memory specificity") OR (Specific phobia AND "overgeneral memory") OR (Specific phobia AND "memory specificity" AND ruminat*) OR (Specific phobia AND "memory specificity" AND brooding) OR (Specific phobia AND "memory specificity" AND reflection) OR (Specific phobia AND "memory specificity" AND "executive function") OR (Specific phobia AND "memory specificity" AND "executive control") OR (Specific phobia AND "memory specificity" AND avoidance) OR (Specific phobia AND "memory specificity" AND "verbal fluency") OR (Specific phobia AND "memory specificity" AND "semantic fluency") OR (Specific phobia AND "memory specificity" AND "inhibitory control") OR (Specific phobia AND "memory specificity" AND updating) OR (Specific phobia AND "memory specificity" AND "repetitive thinking") OR (Specific phobia AND "memory specificity" AND worry) OR (Specific phobia AND "memory specificity" AND "problem solving") OR (Specific phobia AND "memory specificity" AND "future thinking") OR (Specific phobia AND "overgeneral memory" AND ruminat*) OR (Specific phobia AND "overgeneral memory" AND brooding) OR (Specific phobia AND "overgeneral memory" AND reflection) OR (Specific phobia AND "overgeneral memory" AND "executive function") OR (Specific phobia AND "overgeneral memory" AND "executive control") OR (Specific phobia AND "overgeneral memory" AND avoidance) OR (Specific phobia AND "overgeneral memory" AND "verbal fluency") OR (Specific phobia AND "overgeneral memory" AND "semantic fluency") OR (Specific phobia AND "overgeneral memory" AND "inhibitory control") OR (Specific phobia AND "overgeneral memory" AND updating) OR (Specific phobia AND "overgeneral memory" AND "repetitive thinking") OR (Specific phobia AND "overgeneral memory" AND worry) OR (Specific phobia AND "overgeneral memory" AND "problem solving") OR (Specific phobia AND "overgeneral memory" AND "future thinking")

**Substance Abuse Disorder**

(Substance Abuse Disorder AND "memory specificity") OR (Substance Abuse Disorder AND "overgeneral memory") OR (Substance Abuse Disorder AND "memory specificity" AND ruminat*) OR (Substance Abuse Disorder AND "memory specificity" AND brooding) OR (Substance Abuse Disorder AND "memory specificity" AND reflection) OR (Substance Abuse Disorder AND "memory specificity" AND "executive function") OR (Substance Abuse Disorder AND "memory specificity" AND "executive control") OR (Substance Abuse Disorder AND "memory specificity" AND avoidance) OR (Substance Abuse Disorder AND "memory specificity" AND "verbal fluency") OR (Substance Abuse Disorder AND "memory specificity" AND "semantic fluency") OR (Substance Abuse Disorder AND "memory specificity" AND "inhibitory control") OR (Substance Abuse Disorder AND "memory specificity" AND updating) OR (Substance Abuse Disorder AND "memory specificity" AND "repetitive thinking") OR (Substance Abuse Disorder AND "memory specificity" AND worry) OR (Substance Abuse Disorder AND "memory specificity" AND "problem solving") OR (Substance Abuse Disorder AND "memory specificity" AND "future thinking") OR (Substance Abuse Disorder AND "overgeneral memory" AND ruminat*) OR (Substance Abuse Disorder AND "overgeneral memory" AND brooding) OR (Substance Abuse Disorder AND "overgeneral memory" AND reflection) OR (Substance Abuse Disorder AND "overgeneral memory" AND "executive function") OR (Substance Abuse Disorder AND "overgeneral memory" AND "executive control") OR (Substance Abuse Disorder AND "overgeneral memory" AND avoidance) OR (Substance Abuse Disorder AND "overgeneral memory" AND "verbal fluency") OR (Substance Abuse Disorder AND "overgeneral memory" AND "semantic fluency") OR (Substance Abuse Disorder AND "overgeneral memory" AND "inhibitory control") OR (Substance Abuse Disorder AND "overgeneral memory" AND updating) OR (Substance Abuse Disorder AND "overgeneral memory" AND "repetitive thinking") OR (Substance Abuse Disorder AND "overgeneral memory" AND worry) OR (Substance Abuse Disorder AND "overgeneral memory" AND "problem solving") OR (Substance Abuse Disorder AND "overgeneral memory" AND "future thinking")

**Substance use disorder**
(Substance use disorder AND "memory specificity") OR (Substance use disorder AND "overgeneral memory") OR (Substance use disorder AND "memory specificity" AND ruminat*) OR (Substance use disorder AND "memory specificity" AND brooding) OR (Substance use disorder AND "memory specificity" AND reflection) OR (Substance use disorder AND "memory specificity" AND "executive function") OR (Substance use disorder AND "memory specificity" AND "executive control") OR (Substance use disorder AND "memory specificity" AND avoidance) OR (Substance use disorder AND "memory specificity" AND "verbal fluency") OR (Substance use disorder AND "memory specificity" AND "semantic fluency") OR (Substance use disorder AND "memory specificity" AND "inhibitory control") OR (Substance use disorder AND "memory specificity" AND updating) OR (Substance use disorder AND "memory specificity" AND "repetitive thinking") OR (Substance use disorder AND "memory specificity" AND worry) OR (Substance use disorder AND "memory specificity" AND "problem solving") OR (Substance use disorder AND "memory specificity" AND "future thinking") OR (Substance use disorder AND "overgeneral memory" AND ruminat*) OR (Substance use disorder AND "overgeneral memory" AND brooding) OR (Substance use disorder AND "overgeneral memory" AND reflection) OR (Substance use disorder AND "overgeneral memory" AND "executive function") OR (Substance use disorder AND "overgeneral memory" AND "executive control") OR (Substance use disorder AND "overgeneral memory" AND avoidance) OR (Substance use disorder AND "overgeneral memory" AND "verbal fluency") OR (Substance use disorder AND "overgeneral memory" AND "semantic fluency") OR (Substance use disorder AND "overgeneral memory" AND "inhibitory control") OR (Substance use disorder AND "overgeneral memory" AND updating) OR (Substance use disorder AND "overgeneral memory" AND "repetitive thinking") OR (Substance use disorder AND "overgeneral memory" AND worry) OR (Substance use disorder AND "overgeneral memory" AND "problem solving") OR (Substance use disorder AND "overgeneral memory" AND "future thinking")

**Eating Disorder**

(Eating Disorder AND "memory specificity") OR (Eating Disorder AND "overgeneral memory")OR (Eating Disorder AND "memory specificity" AND ruminat*) OR (Eating Disorder AND "memory specificity" AND brooding) OR (Eating Disorder AND "memory specificity" AND reflection) OR (Eating Disorder AND "memory specificity" AND "executive function") OR (Eating Disorder AND "memory specificity" AND "executive control") OR (Eating Disorder AND "memory specificity" AND avoidance) OR (Eating Disorder AND "memory specificity" AND "verbal fluency") OR (Eating Disorder AND "memory specificity" AND "semantic fluency") OR (Eating Disorder AND "memory specificity" AND "inhibitory control") OR (Eating Disorder AND "memory specificity" AND updating) OR (Eating Disorder AND "memory specificity" AND "repetitive thinking") OR (Eating Disorder AND "memory specificity" AND worry) OR (Eating Disorder AND "memory specificity" AND "problem solving") OR (Eating Disorder AND "memory specificity" AND "future thinking") OR (Eating Disorder AND "overgeneral memory" AND ruminat*) OR (Eating Disorder AND "overgeneral memory" AND brooding) OR (Eating Disorder AND "overgeneral memory" AND reflection) OR (Eating Disorder AND "overgeneral memory" AND "executive function") OR (Eating Disorder AND "overgeneral memory" AND "executive control") OR (Eating Disorder AND "overgeneral memory" AND avoidance) OR (Eating Disorder AND "overgeneral memory" AND "verbal fluency") OR (Eating Disorder AND "overgeneral memory" AND "semantic fluency") OR (Eating Disorder AND "overgeneral memory" AND "inhibitory control") OR (Eating Disorder AND "overgeneral memory" AND updating) OR (Eating Disorder AND "overgeneral memory" AND "repetitive thinking") OR (Eating Disorder AND "overgeneral memory" AND worry) OR (Eating Disorder AND "overgeneral memory" AND "problem solving") OR (Eating Disorder AND "overgeneral memory" AND "future thinking")

**Anorexia Nervosa**

(Anorexia Nervosa AND "memory specificity") OR (Anorexia Nervosa AND "overgeneral memory")OR (Anorexia Nervosa AND "memory specificity" AND ruminat*) OR (Anorexia Nervosa AND "memory specificity" AND brooding) OR (Anorexia Nervosa AND "memory specificity" AND reflection) OR (Anorexia Nervosa AND "memory specificity" AND "executive function") OR (Anorexia Nervosa AND "memory specificity" AND "executive control") OR (Anorexia Nervosa AND "memory specificity" AND avoidance) OR (Anorexia Nervosa AND "memory specificity" AND "verbal fluency") OR (Anorexia Nervosa AND "memory specificity" AND "semantic fluency") OR (Anorexia Nervosa AND "memory specificity" AND "inhibitory control") OR (Anorexia Nervosa AND "memory specificity" AND updating) OR (Anorexia Nervosa AND "memory specificity" AND "repetitive thinking") OR (Anorexia Nervosa AND "memory specificity" AND worry) OR (Anorexia Nervosa AND "memory specificity" AND "problem solving") OR (Anorexia Nervosa AND "memory specificity" AND "future thinking") OR (Anorexia Nervosa AND "overgeneral memory" AND ruminat*) OR (Anorexia Nervosa AND "overgeneral memory" AND brooding) OR (Anorexia Nervosa AND "overgeneral memory" AND reflection) OR (Anorexia Nervosa AND "overgeneral memory" AND "executive function") OR (Anorexia Nervosa AND "overgeneral memory" AND "executive control") OR (Anorexia Nervosa AND "overgeneral memory" AND avoidance) OR (Anorexia Nervosa AND "overgeneral memory" AND "verbal fluency") OR (Anorexia Nervosa AND "overgeneral memory" AND "semantic fluency") OR (Anorexia Nervosa AND "overgeneral memory" AND "inhibitory control") OR (Anorexia Nervosa AND "overgeneral memory" AND updating) OR (Anorexia Nervosa AND "overgeneral memory" AND "repetitive thinking") OR (Anorexia Nervosa AND "overgeneral memory" AND worry) OR (Anorexia Nervosa AND "overgeneral memory" AND "problem solving") OR (Anorexia Nervosa AND "overgeneral memory" AND "future thinking")

**Bulimia Nervosa**

(Bulimia Nervosa AND "memory specificity") OR (Bulimia Nervosa AND "overgeneral memory") OR (Bulimia Nervosa AND "memory specificity" AND ruminat*) OR (Bulimia Nervosa AND "memory specificity" AND brooding) OR (Bulimia Nervosa AND "memory specificity" AND reflection) OR (Bulimia Nervosa AND "memory specificity" AND "executive function") OR (Bulimia Nervosa AND "memory specificity" AND "executive control") OR (Bulimia Nervosa AND "memory specificity" AND avoidance) OR (Bulimia Nervosa AND "memory specificity" AND "verbal fluency") OR (Bulimia Nervosa AND "memory specificity" AND "semantic fluency") OR (Bulimia Nervosa AND "memory specificity" AND "inhibitory control") OR (Bulimia Nervosa AND "memory specificity" AND updating) OR (Bulimia Nervosa AND "memory specificity" AND "repetitive thinking") OR (Bulimia Nervosa AND "memory specificity" AND worry) OR (Bulimia Nervosa AND "memory specificity" AND "problem solving") OR (Bulimia Nervosa AND "memory specificity" AND "future thinking") OR (Bulimia Nervosa AND "overgeneral memory" AND ruminat*) OR (Bulimia Nervosa AND "overgeneral memory" AND brooding) OR (Bulimia Nervosa AND "overgeneral memory" AND reflection) OR (Bulimia Nervosa AND "overgeneral memory" AND "executive function") OR (Bulimia Nervosa AND "overgeneral memory" AND "executive control") OR (Bulimia Nervosa AND "overgeneral memory" AND avoidance) OR (Bulimia Nervosa AND "overgeneral memory" AND "verbal fluency") OR (Bulimia Nervosa AND "overgeneral memory" AND "semantic fluency") OR (Bulimia Nervosa AND "overgeneral memory" AND "inhibitory control") OR (Bulimia Nervosa AND "overgeneral memory" AND updating) OR (Bulimia Nervosa AND "overgeneral memory" AND "repetitive thinking") OR (Bulimia Nervosa AND "overgeneral memory" AND worry) OR (Bulimia Nervosa AND "overgeneral memory" AND "problem solving") OR (Bulimia Nervosa AND "overgeneral memory" AND "future thinking")

**Binge-Eating Disorder**

(Binge Eating Disorder AND "memory specificity") OR (Binge Eating Disorder AND "overgeneral memory") OR (Binge Eating Disorder AND "memory specificity" AND ruminat*) OR (Binge Eating Disorder AND "memory specificity" AND brooding) OR (Binge Eating Disorder AND "memory specificity" AND reflection) OR (Binge Eating Disorder AND "memory specificity" AND "executive function") OR (Binge Eating Disorder AND "memory specificity" AND "executive control") OR (Binge Eating Disorder AND "memory specificity" AND avoidance) OR (Binge Eating Disorder AND "memory specificity" AND "verbal fluency") OR (Binge Eating Disorder AND "memory specificity" AND "semantic fluency") OR (Binge Eating Disorder AND "memory specificity" AND "inhibitory control") OR (Binge Eating Disorder AND "memory specificity" AND updating) OR (Binge Eating Disorder AND "memory specificity" AND "repetitive thinking") OR (Binge Eating Disorder AND "memory specificity" AND worry) OR (Binge Eating Disorder AND "memory specificity" AND "problem solving") OR (Binge Eating Disorder AND "memory specificity" AND "future thinking") OR (Binge Eating Disorder AND "overgeneral memory" AND ruminat*) OR (Binge Eating Disorder AND "overgeneral memory" AND brooding) OR (Binge Eating Disorder AND "overgeneral memory" AND reflection) OR (Binge Eating Disorder AND "overgeneral memory" AND "executive function") OR (Binge Eating Disorder AND "overgeneral memory" AND "executive control") OR (Binge Eating Disorder AND "overgeneral memory" AND avoidance) OR (Binge Eating Disorder AND "overgeneral memory" AND "verbal fluency") OR (Binge Eating Disorder AND "overgeneral memory" AND "semantic fluency") OR (Binge Eating Disorder AND "overgeneral memory" AND "inhibitory control") OR (Binge Eating Disorder AND "overgeneral memory" AND updating) OR (Binge Eating Disorder AND "overgeneral memory" AND "repetitive thinking") OR (Binge Eating Disorder AND "overgeneral memory" AND worry) OR (Binge Eating Disorder AND "overgeneral memory" AND "problem solving") OR (Binge Eating Disorder AND "overgeneral memory" AND "future thinking")

**Obsessive Compulsive Disorder**

(Obsessive compulsive disorder AND "memory specificity") OR (Obsessive compulsive disorder AND "overgeneral memory") OR (Obsessive compulsive disorder AND "memory specificity" AND ruminat*) OR (Obsessive compulsive disorder AND "memory specificity" AND brooding) OR (Obsessive compulsive disorder AND "memory specificity" AND reflection) OR (Obsessive compulsive disorder AND "memory specificity" AND "executive function") OR (Obsessive compulsive disorder AND "memory specificity" AND "executive control") OR (Obsessive compulsive disorder AND "memory specificity" AND avoidance) OR (Obsessive compulsive disorder AND "memory specificity" AND "verbal fluency") OR (Obsessive compulsive disorder AND "memory specificity" AND "semantic fluency") OR (Obsessive compulsive disorder AND "memory specificity" AND "inhibitory control") OR (Obsessive compulsive disorder AND "memory specificity" AND updating) OR (Obsessive compulsive disorder AND "memory specificity" AND "repetitive thinking") OR (Obsessive compulsive disorder AND "memory specificity" AND worry) OR (Obsessive compulsive disorder AND "memory specificity" AND "problem solving") OR (Obsessive compulsive disorder AND "memory specificity" AND "future thinking") OR (Obsessive compulsive disorder AND "overgeneral memory" AND ruminat*) OR (Obsessive compulsive disorder AND "overgeneral memory" AND brooding) OR (Obsessive compulsive disorder AND "overgeneral memory" AND reflection) OR (Obsessive compulsive disorder AND "overgeneral memory" AND "executive function") OR (Obsessive compulsive disorder AND "overgeneral memory" AND "executive control") OR (Obsessive compulsive disorder AND "overgeneral memory" AND avoidance) OR (Obsessive compulsive disorder AND "overgeneral memory" AND "verbal fluency") OR (Obsessive compulsive disorder AND "overgeneral memory" AND "semantic fluency") OR (Obsessive compulsive disorder AND "overgeneral memory" AND "inhibitory control") OR (Obsessive compulsive disorder AND "overgeneral memory" AND updating) OR (Obsessive compulsive disorder AND "overgeneral memory" AND "repetitive thinking") OR (Obsessive compulsive disorder AND "overgeneral memory" AND worry) OR (Obsessive compulsive disorder AND "overgeneral memory" AND "problem solving") OR (Obsessive compulsive disorder AND "overgeneral memory" AND "future thinking")

**Body Dysmorphic Disorder**

(Body Dysmorphic Disorder AND "memory specificity") OR (Body Dysmorphic Disorder AND "overgeneral memory")OR (Body Dysmorphic Disorder AND "memory specificity" AND ruminat*) OR (Body Dysmorphic Disorder AND "memory specificity" AND brooding) OR (Body Dysmorphic Disorder AND "memory specificity" AND reflection) OR (Body Dysmorphic Disorder AND "memory specificity" AND "executive function") OR (Body Dysmorphic Disorder AND "memory specificity" AND "executive control") OR (Body Dysmorphic Disorder AND "memory specificity" AND avoidance) OR (Body Dysmorphic Disorder AND "memory specificity" AND "verbal fluency") OR (Body Dysmorphic Disorder AND "memory specificity" AND "semantic fluency") OR (Body Dysmorphic Disorder AND "memory specificity" AND "inhibitory control") OR (Body Dysmorphic Disorder AND "memory specificity" AND updating) OR (Body Dysmorphic Disorder AND "memory specificity" AND "repetitive thinking") OR (Body Dysmorphic Disorder AND "memory specificity" AND worry) OR (Body Dysmorphic Disorder AND "memory specificity" AND "problem solving") OR (Body Dysmorphic Disorder AND "memory specificity" AND "future thinking") OR (Body Dysmorphic Disorder AND "overgeneral memory" AND ruminat*) OR (Body Dysmorphic Disorder AND "overgeneral memory" AND brooding) OR (Body Dysmorphic Disorder AND "overgeneral memory" AND reflection) OR (Body Dysmorphic Disorder AND "overgeneral memory" AND "executive function") OR (Body Dysmorphic Disorder AND "overgeneral memory" AND "executive control") OR (Body Dysmorphic Disorder AND "overgeneral memory" AND avoidance) OR (Body Dysmorphic Disorder AND "overgeneral memory" AND "verbal fluency") OR (Body Dysmorphic Disorder AND "overgeneral memory" AND "semantic fluency") OR (Body Dysmorphic Disorder AND "overgeneral memory" AND "inhibitory control") OR (Body Dysmorphic Disorder AND "overgeneral memory" AND updating) OR (Body Dysmorphic Disorder AND "overgeneral memory" AND "repetitive thinking") OR (Body Dysmorphic Disorder AND "overgeneral memory" AND worry) OR (Body Dysmorphic Disorder AND "overgeneral memory" AND "problem solving") OR (Body Dysmorphic Disorder AND "overgeneral memory" AND "future thinking")

**Psychotic disorder**

(Psychotic disorder AND "memory specificity") OR (Psychotic disorder AND "overgeneral memory") OR (Psychotic disorder AND "memory specificity" AND ruminat*) OR (Psychotic disorder AND "memory specificity" AND brooding) OR (Psychotic disorder AND "memory specificity" AND reflection) OR (Psychotic disorder AND "memory specificity" AND "executive function") OR (Psychotic disorder AND "memory specificity" AND "executive control") OR (Psychotic disorder AND "memory specificity" AND avoidance) OR (Psychotic disorder AND "memory specificity" AND "verbal fluency") OR (Psychotic disorder AND "memory specificity" AND "semantic fluency") OR (Psychotic disorder AND "memory specificity" AND "inhibitory control") OR (Psychotic disorder AND "memory specificity" AND updating) OR (Psychotic disorder AND "memory specificity" AND "repetitive thinking") OR (Psychotic disorder AND "memory specificity" AND worry) OR (Psychotic disorder AND "memory specificity" AND "problem solving") OR (Psychotic disorder AND "memory specificity" AND "future thinking") OR (Psychotic disorder AND "overgeneral memory" AND ruminat*) OR (Psychotic disorder AND "overgeneral memory" AND brooding) OR (Psychotic disorder AND "overgeneral memory" AND reflection) OR (Psychotic disorder AND "overgeneral memory" AND "executive function") OR (Psychotic disorder AND "overgeneral memory" AND "executive control") OR (Psychotic disorder AND "overgeneral memory" AND avoidance) OR (Psychotic disorder AND "overgeneral memory" AND "verbal fluency") OR (Psychotic disorder AND "overgeneral memory" AND "semantic fluency") OR (Psychotic disorder AND "overgeneral memory" AND "inhibitory control") OR (Psychotic disorder AND "overgeneral memory" AND updating) OR (Psychotic disorder AND "overgeneral memory" AND "repetitive thinking") OR (Psychotic disorder AND "overgeneral memory" AND worry) OR (Psychotic disorder AND "overgeneral memory" AND "problem solving") OR (Psychotic disorder AND "overgeneral memory" AND "future thinking")

**Sleep wake disorder**

(Sleep wake disorder AND "memory specificity") OR (Sleep wake disorder AND "overgeneral memory") OR (Sleep wake disorder AND "memory specificity" AND ruminat*) OR (Sleep wake disorder AND "memory specificity" AND brooding) OR (Sleep wake disorder AND "memory specificity" AND reflection) OR (Sleep wake disorder AND "memory specificity" AND "executive function") OR (Sleep wake disorder AND "memory specificity" AND "executive control") OR (Sleep wake disorder AND "memory specificity" AND avoidance) OR (Sleep wake disorder AND "memory specificity" AND "verbal fluency") OR (Sleep wake disorder AND "memory specificity" AND "semantic fluency") OR (Sleep wake disorder AND "memory specificity" AND "inhibitory control") OR (Sleep wake disorder AND "memory specificity" AND updating) OR (Sleep wake disorder AND "memory specificity" AND "repetitive thinking") OR (Sleep wake disorder AND "memory specificity" AND worry) OR (Sleep wake disorder AND "memory specificity" AND "problem solving") OR (Sleep wake disorder AND "memory specificity" AND "future thinking") OR (Sleep wake disorder AND "overgeneral memory" AND ruminat*) OR (Sleep wake disorder AND "overgeneral memory" AND brooding) OR (Sleep wake disorder AND "overgeneral memory" AND reflection) OR (Sleep wake disorder AND "overgeneral memory" AND "executive function") OR (Sleep wake disorder AND "overgeneral memory" AND "executive control") OR (Sleep wake disorder AND "overgeneral memory" AND avoidance) OR (Sleep wake disorder AND "overgeneral memory" AND "verbal fluency") OR (Sleep wake disorder AND "overgeneral memory" AND "semantic fluency") OR (Sleep wake disorder AND "overgeneral memory" AND "inhibitory control") OR (Sleep wake disorder AND "overgeneral memory" AND updating) OR (Sleep wake disorder AND "overgeneral memory" AND "repetitive thinking") OR (Sleep wake disorder AND "overgeneral memory" AND worry) OR (Sleep wake disorder AND "overgeneral memory" AND "problem solving") OR (Sleep wake disorder AND "overgeneral memory" AND "future thinking")

**Insomnia**

(Insomnia AND "memory specificity") OR (Insomnia AND "overgeneral memory") OR (Insomnia AND "memory specificity" AND ruminat*) OR (Insomnia AND "memory specificity" AND brooding) OR (Insomnia AND "memory specificity" AND reflection) OR (Insomnia AND "memory specificity" AND "executive function") OR (Insomnia AND "memory specificity" AND "executive control") OR (Insomnia AND "memory specificity" AND avoidance) OR (Insomnia AND "memory specificity" AND "verbal fluency") OR (Insomnia AND "memory specificity" AND "semantic fluency") OR (Insomnia AND "memory specificity" AND "inhibitory control") OR (Insomnia AND "memory specificity" AND updating) OR (Insomnia AND "memory specificity" AND "repetitive thinking") OR (Insomnia AND "memory specificity" AND worry) OR (Insomnia AND "memory specificity" AND "problem solving") OR (Insomnia AND "memory specificity" AND "future thinking") OR (Insomnia AND "overgeneral memory" AND ruminat*) OR (Insomnia AND "overgeneral memory" AND brooding) OR (Insomnia AND "overgeneral memory" AND reflection) OR (Insomnia AND "overgeneral memory" AND "executive function") OR (Insomnia AND "overgeneral memory" AND "executive control") OR (Insomnia AND "overgeneral memory" AND avoidance) OR (Insomnia AND "overgeneral memory" AND "verbal fluency") OR (Insomnia AND "overgeneral memory" AND "semantic fluency") OR (Insomnia AND "overgeneral memory" AND "inhibitory control") OR (Insomnia AND "overgeneral memory" AND updating) OR (Insomnia AND "overgeneral memory" AND "repetitive thinking") OR (Insomnia AND "overgeneral memory" AND worry) OR (Insomnia AND "overgeneral memory" AND "problem solving") OR (Insomnia AND "overgeneral memory" AND "future thinking")

**Personality Disorder**

(Personality Disorder AND "memory specificity") OR (Personality Disorder AND "overgeneral memory") OR (Personality Disorder AND "memory specificity" AND ruminat*) OR (Personality Disorder AND "memory specificity" AND brooding) OR (Personality Disorder AND "memory specificity" AND reflection) OR (Personality Disorder AND "memory specificity" AND "executive function") OR (Personality Disorder ND "memory specificity" AND "executive control") OR (Personality Disorder AND "memory specificity" AND avoidance) OR (Personality Disorder AND "memory specificity" AND "verbal fluency") OR (Personality Disorder AND "memory specificity" AND "semantic fluency") OR (Personality Disorder AND "memory specificity" AND "inhibitory control") OR (Personality Disorder AND "memory specificity" AND updating) OR (Personality Disorder AND "memory specificity" AND "repetitive thinking") OR (Personality Disorder AND "memory specificity" AND worry) OR (Personality Disorder AND "memory specificity" AND "problem solving") OR (Personality Disorder AND "memory specificity" AND "future thinking") OR (Personality Disorder AND "overgeneral memory" AND ruminat*) OR (Personality Disorder AND "overgeneral memory" AND brooding) OR (Personality Disorder AND "overgeneral memory" AND reflection) OR (Personality Disorder AND "overgeneral memory" AND "executive function") OR (Personality Disorder AND "overgeneral memory" AND "executive control") OR (Personality Disorder AND "overgeneral memory" AND avoidance) OR (Personality Disorder AND "overgeneral memory" AND "verbal fluency") OR (Personality Disorder AND "overgeneral memory" AND "semantic fluency") OR (Personality Disorder AND "overgeneral memory" AND "inhibitory control") OR (Personality Disorder AND "overgeneral memory" AND updating) OR (Personality Disorder AND "overgeneral memory" AND "repetitive thinking") OR (Personality Disorder AND "overgeneral memory" AND worry) OR (Personality Disorder AND "overgeneral memory" AND "problem solving") OR (Personality Disorder AND "overgeneral memory" AND "future thinking")

**Sexual Disorder**

(Sexual Disorder AND "memory specificity") OR (Sexual Disorder AND "overgeneral memory")OR (Sexual Disorder AND "memory specificity" AND ruminat*) OR (Sexual Disorder AND "memory specificity" AND brooding) OR (Sexual Disorder AND "memory specificity" AND reflection) OR (Sexual Disorder AND "memory specificity" AND "executive function") OR (Sexual Disorder AND "memory specificity" AND "executive control") OR (Sexual Disorder AND "memory specificity" AND avoidance) OR (Sexual Disorder AND "memory specificity" AND "verbal fluency") OR (Sexual Disorder AND "memory specificity" AND "semantic fluency") OR (Sexual Disorder AND "memory specificity" AND "inhibitory control") OR (Sexual Disorder AND "memory specificity" AND updating) OR (Sexual Disorder AND "memory specificity" AND "repetitive thinking") OR (Sexual Disorder AND "memory specificity" AND worry) OR (Sexual Disorder AND "memory specificity" AND "problem solving") OR (Sexual Disorder AND "memory specificity" AND "future thinking") OR (Sexual Disorder AND "overgeneral memory" AND ruminat*) OR (Sexual Disorder AND "overgeneral memory" AND brooding) OR (Sexual Disorder AND "overgeneral memory" AND reflection) OR (Sexual Disorder AND "overgeneral memory" AND "executive function") OR (Sexual Disorder AND "overgeneral memory" AND "executive control") OR (Sexual Disorder AND "overgeneral memory" AND avoidance) OR (Sexual Disorder AND "overgeneral memory" AND "verbal fluency") OR (Sexual Disorder AND "overgeneral memory" AND "semantic fluency") OR (Sexual Disorder AND "overgeneral memory" AND "inhibitory control") OR (Sexual Disorder AND "overgeneral memory" AND updating) OR (Sexual Disorder AND "overgeneral memory" AND "repetitive thinking") OR (Sexual Disorder AND "overgeneral memory" AND worry) OR (Sexual Disorder AND "overgeneral memory" AND "problem solving") OR (Sexual Disorder AND "overgeneral memory" AND "future thinking")
